# ALKBH5‐mediated m6A modification of lncRNA KCNQ1OT1 triggers the development of LSCC via upregulation of HOXA9

**DOI:** 10.1111/jcmm.17091

**Published:** 2021-12-01

**Authors:** Yushan Li, Bingrui Yan, Xin Wang, Qiuying Li, Xuan Kan, Jingting Wang, Yanan Sun, Peng Wang, Linli Tian, Ming Liu

**Affiliations:** ^1^ Department of Otorhinolaryngology, Head and Neck Surgery The Second Affiliated Hospital Harbin Medical University Harbin China

**Keywords:** ALKBH5, HOXA9, laryngeal squamous cell cancer, lncRNA KCNQ1OT1, m6A methylation

## Abstract

It has been shown that N6‐methyladenosine (m6A) modification is involved in the development of complex human diseases, especially in the development of cancer. Our research investigated the role and mechanism of the m6A modification of lncRNA KCNQ1 overlapping transcript 1 (KCNQ1OT1) in Laryngeal squamous cell carcinoma (LSCC) progression. Microarray analysis was used to quantitatively detect the m6A apparent transcriptional modification level of lncRNA in LSCC tissue. Methylated RNA immunoprecipitation‐qPCR (MeRIP‐qPCR), in situ hybridization (ISH) and quantitative real‐time PCR (qRT‐PCR) were used to examine the m6A modification and expression of KCNQ1OT1. In addition, in vivo and in vitro experiments have tested the effects of KCNQ1OT1 knockdown on the proliferation, invasion and metastasis of LSCC. Mechanically, we found the N6‐methyladenosine (m6A) demethylase ALKBH5 mediates KCNQ1OT1 expression via an m6A‐YTHDF2‐dependent manner and KCNQ1OT1 could directly bind to HOXA9 to further regulate the proliferation, invasion and metastasis of LSCC cells. In general, our research indicates that ALKBH5‐mediated m6A modification of KCNQ1OT1 triggers the development of LSCC via upregulation of HOXA9.

## 1 INTRODUCTION

Laryngeal squamous cell carcinoma (LSCC) is the most common pathological subtype (>95%) of laryngeal carcinoma.^1^, ^2^ The prevalence of LSCC was ranked as 2nd in head and neck squamous cell carcinoma (HNSCC). It is reported that in 2018, there were 177,422 new cases of LSCC and 94,771 deaths of LSCC in the world.^3^ Notably, the incidence of LSCC in China is about four times that in the United States, with an estimated death toll of more than 15,000 per year.^4^ The 5‐year overall survival (OS) rate of patients with advanced LSCC is lower than 50%.^5^ Therefore, we urgently need to have a deeper understanding of the mechanism that promotes LSCC progression.

N6‐methyladenosine (m6A) as a major internal modification of RNA in higher eukaryotes has aroused great interest in recent years.^6^, ^7^, ^8^ The researchers suggested that the m6A methylation plays an important role in the regulation of gene expression by influencing RNA stability, mRNA degradation and translation.^9^, ^10^ This modification mainly depends on the dynamic regulation of RNA methyltransferases (writers), demethylases (erasers) and m6A‐binding proteins (readers).^11^, ^12^ The core components of RNA methyltransferase complex include methyltransferase‐like 3 (METTL3), methyltransferase‐like 14 (METTL14) and Wilms tumour 1‐associated protein (WTAP).^13^ In turn, fat mass and obesity‐associated protein (FTO) and AlkB homolog 5 (ALKBH5) can remove the m6A modification of RNA.^14^ In addition, m6A‐binding proteins, including YTHDF1, YTHDF2, YTHDF3 and YTHDC1, have been identified as m6A modified ‘readers’ and regulate the processing, translation and degradation of mRNA.^15^

Long non‐coding RNA (lncRNA) is an endogenous transcription RNA molecule with a length of more than 200 nucleotides.^16^ More and more studies have proved that lncRNAs play a crucial role in the multi‐steps of tumour development.^17^ For instance, lncRNA growth arrest‐specific 5 (GAS5) suppresses LSCC progression through the negative regulation of miR‐21 and its targets involved in cell proliferation and apoptosis.^18^ Our previous research has demonstrated that lncRNA nuclear paraspeckle assembly transcript 1 (NEAT1) regulates CDK6 expression mediated by miR‐107 in LSCC, which may be a potential target for therapeutic intervention of LSCC.^19^ It has been proved that m6A‐methylated level can affect the expression of lncRNA, and regulate the development of tumour.^20^, ^21^, ^22^

Here, we found that KCNQ1OT1 was a valuable prognostic predictor of LSCC patients and revealed the increase of ALKBH5 can promote the development of LSCC in a m6A‐YTHDF2‐dependent manner by promoting KCNQ1OT1‐HOXA9 signalling. ALKBH5‐KCNQ1OT1‐HOXA9 may be a promising target for the treatment of LSCC.

## 2 METHODS

### 2.1 Patients and specimens

About 86 pairs of LSCC and adjacent non‐tumour tissues were collected from patients who received partial or total laryngectomy in the otolaryngology department of the Second Affiliated Hospital of Harbin Medical University from December 2017 to June 2019. These patients in the study did not receive any cancer treatment before admission. Differentially m6A‐methylated lncRNAs in three pairs of LSCC and non‐tumour tissues were detected by microarray assay, and the level of m6A modification in the top four low m6A methylation modified lncRNAs was detected by MeRIP‐qPCR. In addition, we also detected the expression of ALKBH5 and HOXA9 in 86 pairs of LSCC and non‐tumour tissues by IHC and qRT‐PCR. This study was approved by the ethics committee of Harbin Medical University and obtained informed consent.

### 2.2 Microarray analysis

The Arraystar Human m6A‐lncRNA Epitranscriptomic microarray analysis was from Arraystar Arraystar Company (Rockville, MD, USA). Total RNA from each sample was quantified using the NanoDrop ND‐1000. Briefly, the total RNAs were immunoprecipitated with anti‐N6‐methyladenosine (m6A) antibody. The modified RNAs were eluted from the immunoprecipitated magnetic beads as the ‘IP’. The unmodified RNAs were recovered from the supernatant as ‘Sup’. The ‘IP’ and ‘Sup’ RNAs were labelled with Cy5 and Cy3, respectively, as cRNAs in separate reactions using Arraystar Super RNA Labeling Kit. The cRNAs were combined together and hybridized onto Arraystar Human lncRNA Epitranscriptomic Microarray (8x60K, Arraystar). After washing the slides, the arrays were scanned in two‐colour channels by an Agilent Scanner G2505C.

### 2.3 Methylated RNA immunoprecipitation‐qPCR (MeRIP‐qPCR)

About 1–3 μg of total RNA and m6A labelled control mixture was added into 1ip buffer containing 2 μg of anti‐m6A rabbit polyclonal antibody (synaptic systems, 202003). About 20 μl Dynabeads were incubated at 4°C for 2 h. The suspension of M‐280 Sheep anti‐rabbit IgG (11203d) was sealed at 4°C for 2 h with newly prepared 0.5% BSA. Washed with 300 μl 1×IP buffer for three times, then resuspended in the prepared total RNA antibody mixture, and rotated the RNA bound to the antibody beads for 2 h at 4°C. The beads were then washed three times with 500 μl 1×IP buffer and twice with 500 μl washing buffer. The enriched RNA was eluted with 200 μl elution buffer at 50°C for 1 h. RNA was extracted by acidic phenol chloroform and ethanol precipitation. Then qRT‐PCR detection was performed.

### 2.4 Quantitative real‐time PCR (qRT‐PCR)

According to the manufacturer's protocol, total RNAs was isolated from LSCC samples and cell lines using Trizol Reagents (Invitrogen, Carlsbad, CA, USA). The purified RNA was performed by the Reverse Transcription Kit (Takara, China) to reverse‐transcribed into cDNA. The expression level of RNAs was quantified using the SYBR Green Master Mix (Roche, Switzerland) and normalized to internal control GAPDH mRNA, finally, the 2^−ΔΔCt^ method was executed to detect the relative RNA expression level of RNAs. Three independent experiments were carried out. All primers’ sequences used in the qRT‐PCR were shown in Table S1.

### 2.5 Subcellular fractionation location

According to the manufacturer's instructions, the PARIS Kit (Life Technologies, USA) is used to separate cytoplasmic and nuclear components.

### 2.6 In situ hybridization

ISH was performed to detect the expression of KCNQ1OT1. The RNA scope^®^ 2.5 Assay and HybEZ™ Hybridization System employing in ISH, as well as KCNQ1OT1 target, positive and negative control probes, were provided by Advanced Cell Diagnostics (ACD). The common housekeeping protein, peptidyl‐prolyl isomerase B (PPIB) and the bacterial protein dihydrodipicolinate reductase (DapB) were used as positive and negative control probes, respectively. Slices were scored microscopically according to the manufacturer's instruction. The low expression of KCNQ1OT1 is represented by 0 and 1 scores, while 2, 3 and 4 scores indicated the high expression of KCNQ1OT1.

### 2.7 Cell culture and cell transfection

Human oral keratinocytes (HOK) and human laryngeal cancer cells (TU212 and AMC‐HN‐8) were provided by Tongpai (Shanghai) Biotechnology Co. Ltd. (Shanghai, China), all of which were cultivated conventionally in DMEM medium (HyClone) containing 10% FBS (Biological Industries) and penicillin‐streptomycin (Solaibao) in a humidified incubator at 37°C with an atmosphere with 5% CO2 in air (Thermo Fisher). Lentiviruses knocking down KCNQ1OT1, YTHDF2, ALKBH5, HOXA9 and lentiviruses overexpressing ALKBH5, HOXA9 as well as respective negative control vectors were designed by Genechem Co. Ltd. (Shanghai, China). We inoculated LSCC cells in 6‐well plates for 24h and infected with lentiviruses carrying vectors with negative control, shKCNQ1OT1‐1 and shKCNQ1OT1‐2 constructs for another 24 h. Then, after culturing in DMEM medium containing 2 μg/ml puromycin for 72 h, we used DMEM medium containing 1 μg/ml puromycin to culture cells and generated stably transfected cells. The sequences of shRNAs were shown in Table S2.

### 2.8 Cell proliferation assay

According to the cell counting kit 8 (CCK‐8, Sigma‐Aldrich, MO, US) manufacturer's instructions, 10 μl CCK‐8 solution was added into and incubated with cells. At the appointed time, the absorbance at 450nm was measured. In addition, a 5‐ethynyl‐20‐deoxyuridine assay (EdU) kit (BeyoClick™ EdU Cell Proliferation Kit with Alexa Fluor 555; Shanghai, China) was used for detection of cell proliferation. Every step was carried out in strict accordance with the instructions of the kit.

### 2.9 Colony formation assay

The differently grouped cells were seeded and cultured in 6‐well plate for 2–3 weeks. Then, 4% paraformaldehyde was used to fix cell colony for 15 min, then 0.1% crystal violet was stained for 20–30 min. Images of colony formation were saved and analysed.

### 2.10 Transwell and wound healing assay

Cells were cultured in serum‐free medium for 24 h and resuspended in serum‐free medium at a density of 1 × 10^5^ cells/ml. About 200 μl of cell suspension was added into each Transwell chamber coated with diluted Matrigel (BD Biosciences), as well as, 600 μl of high glucose medium containing 20% FBS was added into each well of 24‐well culture plate. After incubated at 37°C for 24 h, the invaded cells were fixed with 4% paraformaldehyde, stained with 0.1% crystal violet and counted by the microscope in five randomly selected fields. Cells were seeded into a 6‐well plate and incubated to 80% confluence. Vertical scratches in the central area of monolayer adherent cells per well were generated using 100 μl pipette tip. Images of wound of same observed location at different time points were captured by the microscope, and wound healing extent was evaluated by Image J.

### 2.11 In vivo experiment

Animal experiments were carried out with the approval of the Animal Ethics Committee. 4–5 weeks old, 15–20 g weight Balb/c male nude mice were provided by Vital River Laboratory Animal Technology Co. Ltd. (Beijing, China). 1 × 10^6^ (100 µl) cells of infected and uninfected by lentiviral were, respectively, injected subcutaneously into nude mice which divided randomly into scramble group and shKCNQ1OT1‐1 group. After tumour formation, tumour volumes were measured twice a week until mice were euthanized and tumours were surgically removed.

### 2.12 Immunohistochemistry (IHC)

Paraffin‐embedded LSCC and non‐tumour tissues, as well as xenograft tumours, were cutted into 4‐μm slices. The antigen retrieval was performed after samples were removed from paraffin for deparaffinized and rehydrated. The expression levels of Ki‐67, MMP‐2, MMP‐9, HOXA9 and ALKBH5 were measured by immunohistochemical staining with antibodies against Ki‐67 (1:1000) (Abcam, UK), MMP‐2 (1:250) (Abcam, UK), MMP‐9 (1:1000) (Abcam, UK), HOXA9 (1:500) (PL Laboratories), ALKBH5 (1:500) (Abcam, UK). After incubation with primary antibody at 4°C overnight, slices were incubated with secondary antibody for 30 min at room temperature. Slices were then stained with diaminobenzidine and counterstained with haematoxylin. Thereafter, images of IHC staining were obtained by a microscope.

### 2.13 RNA immunoprecipitation (RIP)

The Magna RIP™ RNA‐Binding Protein Immunoprecipitation Kit (Millipore Sigma, 17–700) is used for RNA immunoprecipitation. According to the manufacturer's instructions, RIP lysis buffer was prepared to treat cells. The cell lysates were incubated with protein antibodies and normal rabbit IgG overnight at 4°C. The RNA‐protein/antibody complexes were then immunoprecipitated with protein A/G magnetic beads. RNA is extracted from the precipitated complex for qRT‐PCR.

### 2.14 RNA pull‐down assay and Western blot analysis

In simple terms, biotin (Bio)‐labelled lncRNA KCNQ1OT1 were used to incubate with total proteins from AMC‐HN‐8 and TU212 cell lysates. The complexes formed bound to Streptavidin‐coupled Dynabeads, after which Western blot analysis was carried out to verify the enriched proteins after elution and recovery. After the proteins were extracted and the protein concentration was measured, the proteins were separated electrophoretically in SDS‐PAGE gels and transferred to PVDF membranes. Subsequently, the primary and secondary antibodies were incubated with PVDF membranes, respectively, according to conventional methods. Finally, chemiluminescence detection reagent (ECL) (Solaibao, Beijing, China) was used for detection. All antibodies used in Western blot analysis, unless specially stated, were purchased from Aibokang (Shanghai) Trading Co. Ltd.

### 2.15 RNA stability assays

Medium containing actinomycin D (a9415, Sigma, USA, 5 μg/ml) was used to culture treated cells, and the mRNA expression was calculated. The procedure of isolating total RNA for qPCR analysis was as described previously.

### 2.16 Luciferase reporter assay

AMC‐HN‐8 and TU212 cells were inoculated into 12‐well plates at 50% density. Luciferase reporter gene plasmid and control plasmid were co‐transfected into cells after reaching 70% cell confluency. Following the co‐transfection for 36 h, the Dual‐Luciferase Reporter System (Promega, USA) was used to detect the luciferase activities in cell lysates. The experiment was performed in triplicate.

### 2.17 Statistical analysis

The experiment data were analysed using SPSS version 17.0 software and presented as mean ± SD. All graphs were drawn with GraphPad Prism 5 and 8 software. *P*‐value was calculated by the Student's t tests. *P*‐value with * indicates statistically significant. Three levels of significance (*P < 0.05; ** *P* < 0.01; and ***P < 0.001) were used for all the tests.

## 3 RESULTS

### 3.1 Overview of the m6A‐lncRNA expression profiles in LSCC

**FIGURE 1:**
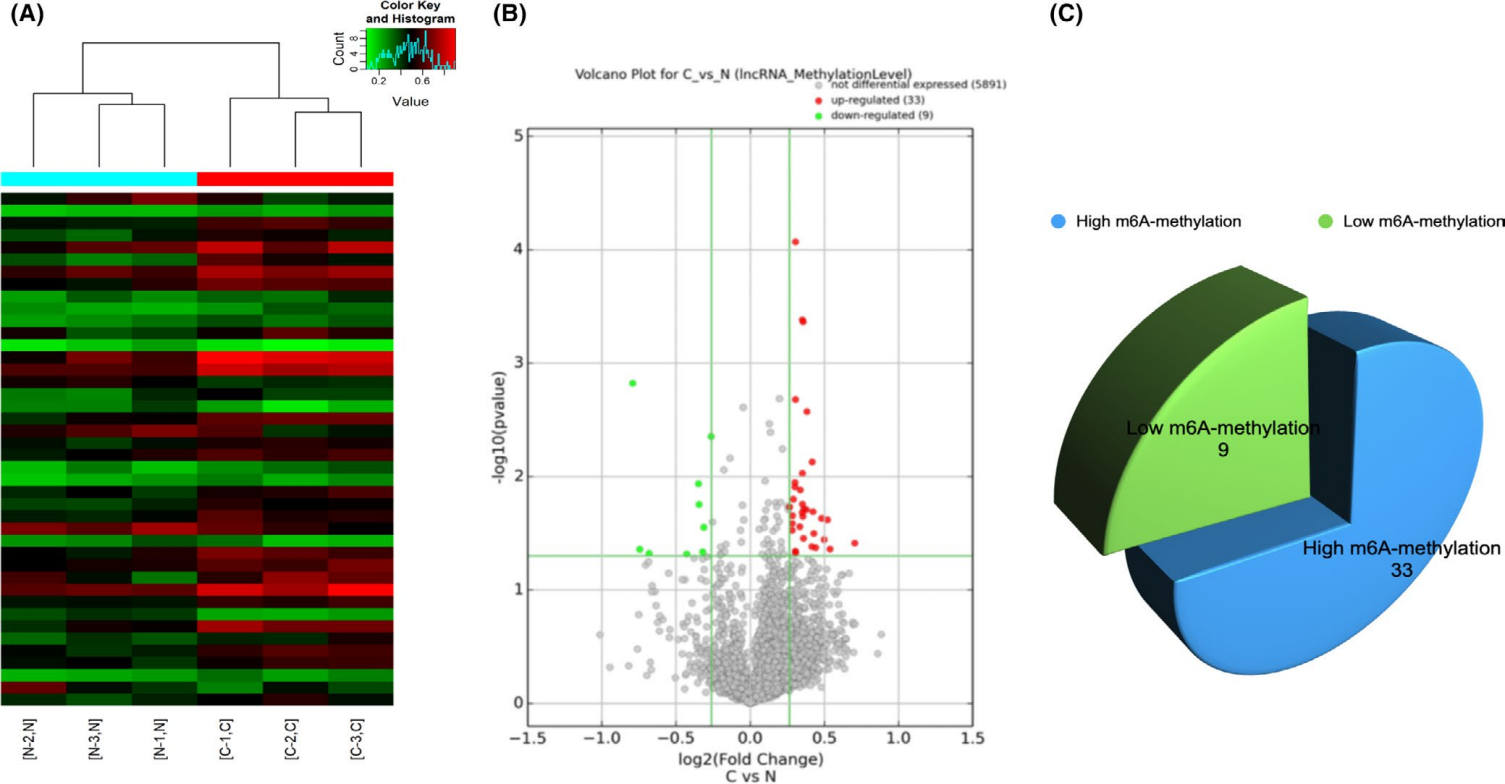
Results of the m6A‐lncRNA expression profiles in LSCC. (A) Hierarchical clustering for lncRNAs with differential ‘m6A quantity’. The red‐green gradient colour scheme indicates the high and low m6A methylation relative quantity as referenced in the Color Key. The top dendrogram shows the tightness between the samples. Sample group members are represented by colour bars above the heat map. (B) Volcano plot. X‐axis: log2 (fold change); Y‐axis: ‐log10 (*P*‐value). The vertical green lines correspond to 1.2‐fold up and down, and the horizontal green line represents 0.05 *p*‐value. The red dot in the figure represents the high m6A methylation lncRNAs, and the green dot represents the low m6A methylation lncRNAs. (C) Pie chart shows 33 high m6A methylation lncRNAs and 9 low m6A methylation lncRNAs

Using the method of Epitranscriptomic Microarray, we evaluated the m6A‐lncRNA expression profiles in three pairs of LSCC and non‐tumour tissues. Differentially m6A‐methylated lncRNAs based on ‘m6A methylation level’ and ‘m6A quantity’ passing fold change and statistical significance cut‐offs were identified and compiled. The default thresholds are |FC| ≥ 1.2 and *p*‐values ≤ 0.05. Hierarchical clustering heatmap analysis was performed for differentially m6A‐methylated lncRNAs. Results of hierarchical clustering heatmap showed that there was a significant m6A‐methylated lncRNAs expression profile between the samples (Figure 1A). Volcanic map showed the significant difference m6A‐methylated lncRNA in LSCC and non‐tumour tissues (Figure 1B). The data showed the significant differential m6A modification of the 42 lncRNAs. 33 lncRNAs showed high m6A methylation and 9 lncRNAs showed low m6A methylation (Figure 1C and Table 1).

**TABLE 1:** Top 18 differently m6A‐methylated lncRNAs

Gene symbol	Fold change	Regulation	Chromsome	Length	*p*‐value
AC004562.1	1.629	Up	chr17	665	0.039
RP11‐426A6.5	1.451	Up	chr9	1117	0.003
LINC00469	1.434	Up	chr17	472	0.024
LOC729683	1.412	Up	chr17	2348	0.036
CTA−293F17.1	1.395	Up	chr7	737	0.023
LOC100131347	1.357	Up	chr17	2305	0.042
RPL7	1.346	Up	chr8	534	0.032
LOC105374727	1.340	Up	chr5	431	0.020
CHST12	1.335	Up	chr7	2067	0.007
KCNQ1OT1	1.732	Down	chr11	91,671	0.048
SPATA6L	1.676	Down	chr9	3435	0.044
RP11‐150O12.5	1.604	Down	chr8	847	0.047
MECP2	1.346	Down	chrX	342	0.002
AE000661.50	1.274	Down	chr14	457	0.012
SELO	1.270	Down	chr22	1626	0.018
RP11‐661A12.7	1.249	Down	chr8	431	0.046
ATP7A	1.242	Down	chrX	5549	0.028
AP000688.8	1.2	Down	chr21	1048	0.004

### 3.2 LncRNA KCNQ1OT1 was low m6A methylation and upregulated in LSCC

**FIGURE 2:**
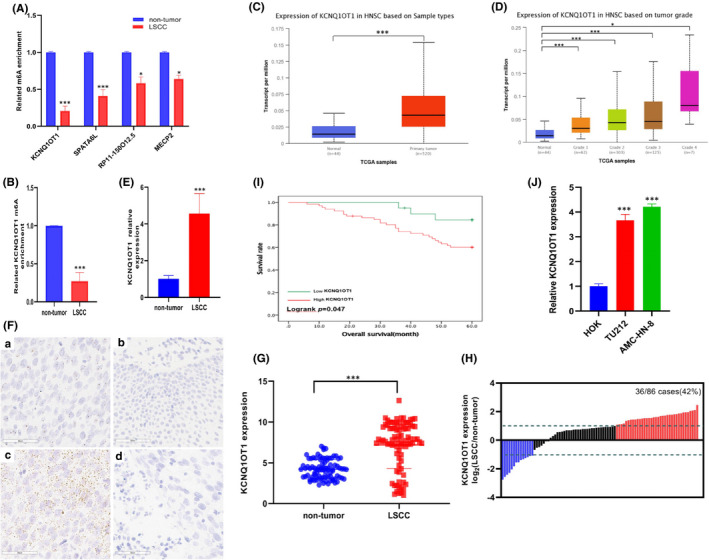
KCNQ1OT1 is low m6A methylation and highly expressed in LSCC. (A) MeRIP‐qPCR was used to verify the level of m6A modification in KCNQ1OT1, SPATA6L, RP11‐150O12.5 and MECP2. (B) MeRIP‐qPCR showed that KCNQ1OT1 was significantly low m6A methylation in the expanded LSCC sample. (C‐D) According to TCGA database, KCNQ1OT1 was overexpressed in HNSCC, and its expression level was related to tumour grade. (E) qRT‐PCR was used to detect the expression of KCNQ1OT1 in the remaining samples. (F) The expression level of KCNQ1OT1 in 86 pairs of LSCC and non‐tumour tissues were investigated by ISH. (a) LSCC tissue; (b) Adjacent tissue; (c) Positive control; (d) Negative control. (G‐H) In the expanded LSCC sample, qRT‐PCR method was used to prove that KCNQ1OT1 was significantly upregulated. (I) KCNQ1OT1 upregulation was significantly associated with poor prognosis. (J) qRT‐PCR showed that KCNQ1OT1 expression in LSCC cell lines was significantly higher than that in HOK cell line

For the remaining samples of the m6A‐lncRNA Epitranscriptomic Microarray, we evaluated the level of m6A modification in KCNQ1OT1, SPATA6L, RP11‐150O12.5 and MECP2 by MeRIP‐qPCR. Compared with non‐tumour tissues, only KCNQ1OT1 was significantly low m6A methylation in LSCC (Figure 2A). Then, we further expanded the LSCC sample to detect KCNQ1OT1 methylation level in 86 pairs of LSCC and non‐tumour tissues. The results of MeRIP‐qPCR showed that KCNQ1OT1 was significantly low m6A methylation in LSCC compared with that in non‐tumour tissues (Figure 2B). Based on the sequence of KCNQ1OT1, we used online prediction software RNA coding potential assessment tool (CPAT) (http://lilab.research.bcm.edu/cpat/index.php) to predict the potential protein‐coding capacity of KCNQ1OT1 and showed KCNQ1OT1 is a non‐coding RNA (Figure S1). Based on TCGA database, we found that KCNQ1OT1 was overexpression in HNSCC and related to tumour grade (Figure 2C,D). Then, KCNQ1OT1 expression was examined in the remaining samples of the m6A‐lncRNA Epitranscriptomic Microarray. The results indicated that the expression of KCNQ1OT1 was remarkable upregulated in the remaining samples (Figure 2E). We also found KCNQ1OT1 was mainly located in the nucleus of LSCC cells (Figure S2). ISH was performed in 86 pairs of LSCC and non‐tumour tissues to verify the level of KCNQ1OT1 was significantly increased in LSCC (Figure 2F). Next, we verified KCNQ1OT1 upregulation in 86 pairs of LSCC and non‐tumour tissues by qRT‐PCR. In this sample cohort, KCNQ1OT1 was significantly upregulated in 42% (36/86) of LSCC patients (Figure 2G,H). According to the correlation between clinicopathological status and KCNQ1OT1 expression in LSCC patients, it was found that the expression of KCNQ1OT1 was correlated with differentiation, smoking and lymph node status (Table 2). Besides, we compared the difference of survival rate between patients with high and low expression levels of KCNQ1OT1 survival curve (86 patients, 5 patients were lost to follow‐up). The results showed that high expression of KCNQ1OT1 was significantly associated with poor prognosis at 60 months (Logrank *p* = 0.047, Figure 2I). The KCNQ1OT1 expression was detected in TU212 and AMC‐HN‐8 cells with HOK cell as negative control. The results showed the KCNQ1OT1 expression in LSCC cell lines was significantly higher (Figure 2J). In conclusion, KCNQ1OT1 is highly expressed and low m6A methylation in LSCC.

**TABLE 2:** Clinical characteristics of 86 LSCC patients according to KCNQ1OT1 expression levels

Characteristics	*n*	KCNQ1OT1 expression		*p*
		Low expression	High expression	
Gender				
Male	62	15	47	0.741
Female	24	5	19	
Age(years)				
<60	40	10	30	0.721
≥60	46	10	36	
T classification				
T1‐2	68	17	51	0.457
T3‐4	18	3	15	
Differentiation				
Well to moderate	66	19	47	0.027*
Poor	20	1	19	
Lymph node metastasis				
Negative	57	18	39	0.010*
Positive	29	2	27	
Primary location				
Supraglottic	42	9	33	0.695
Glottic	44	11	33	
Smoking				
Yes	45	17	28	<0.001***
No	41	3	38	
Family history of cancer				
Yes	54	13	41	0.816
No	32	7	25	

*P*‐value with * indicates statistically significant. Three levels of significance (*P < 0.05; ** *P* < 0.01; and ***P < 0.001) were used for all the tests.

### 3.3 LncRNA KCNQ1OT1 depletion inhibits cell proliferation, migration and invasion of LSCC Cells

**FIGURE 3:**
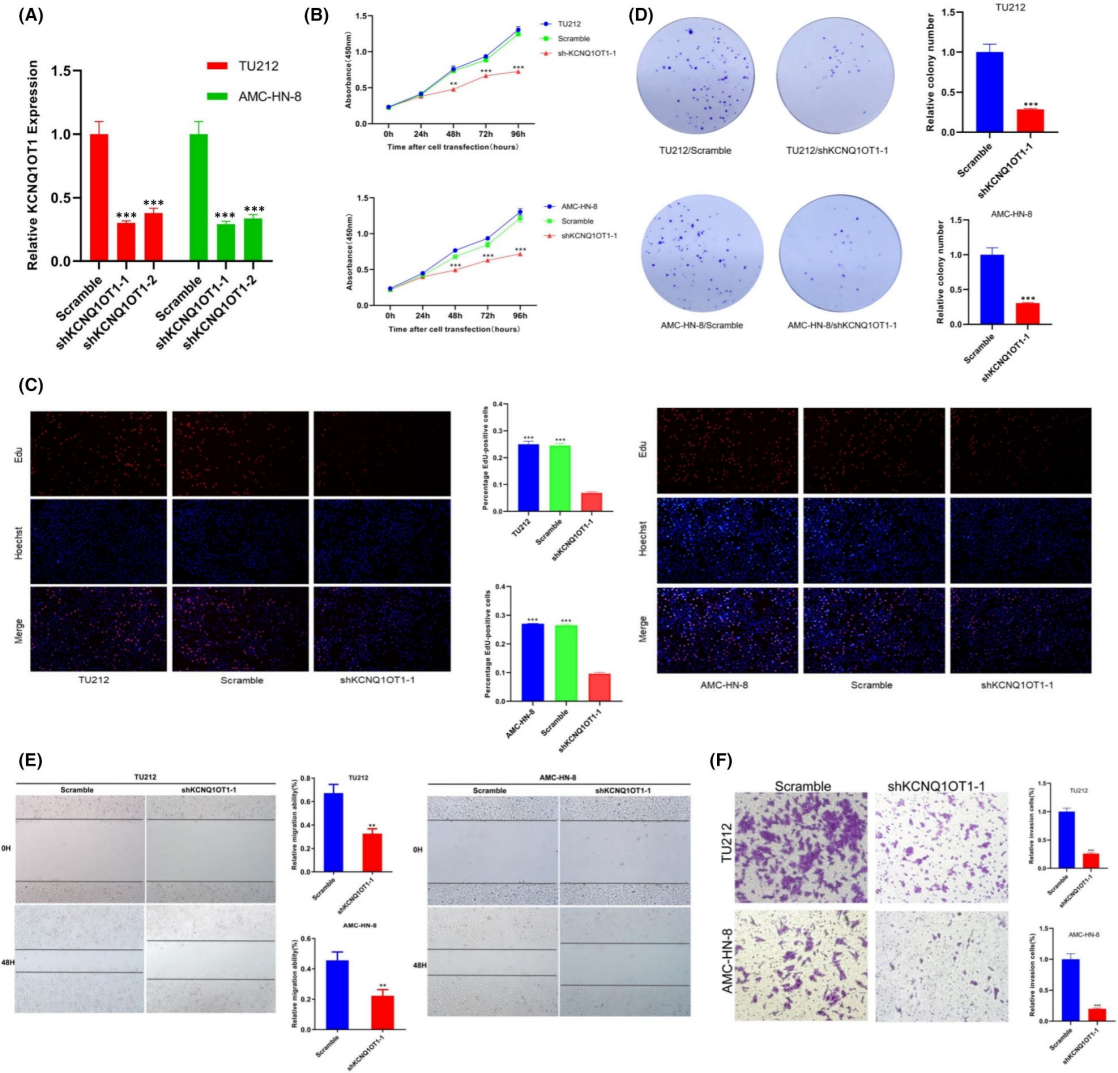
Knockdown of KCNQ1OT1 significantly decreased LSCC cells proliferation, colony formation, migration and invasion. (A) The shKCNQ1OT1‐mediated KCNQ1OT1 repression was confirmed by qRT‐PCR after lentivirus infection in TU212 and AMC‐HN‐8 cells. (B) and (C) showed that KCNQ1OT1 knockdown significantly reduced the cell proliferation rate, as measured by the CCK‐8, Edu. (D) The ability of colony formation was weakened after KCNQ1OT1 knockdown. Knockdown of KCNQ1OT1 decreased wound healing and invasion abilities of LSCC cells in (E) and (F)

Next, we knockdown the expression of KCNQ1OT1 in TU212 and AMC‐HN‐8 cells. ShKCNQ1OT1‐1 was selected for further experiments based on its more effective inhibition (Figure 3A). CCK‐8 and Edu results showed that the growth of LSCC cells was significantly inhibited after KCNQ1OT1 was silenced (Figure 3B,C). Cell staining with crystal violet showed that TU212 and AMC‐HN‐8 cell clone numbers were significantly reduced in the shKCNQ1OT1‐1 group (Figure 3D), which showed that KCNQ1OT1 knockdown inhibited cell proliferation. Furthermore, KCNQ1OT1 knockdown greatly inhibited wound closure and invasive abilities (Figure 3E,F). These results indicate that KCNQ1OT1 promotes the growth of LSCC in vitro.

### 3.4 KCNQ1OT1 promotes LSCC growth in vivo

**FIGURE 4:**
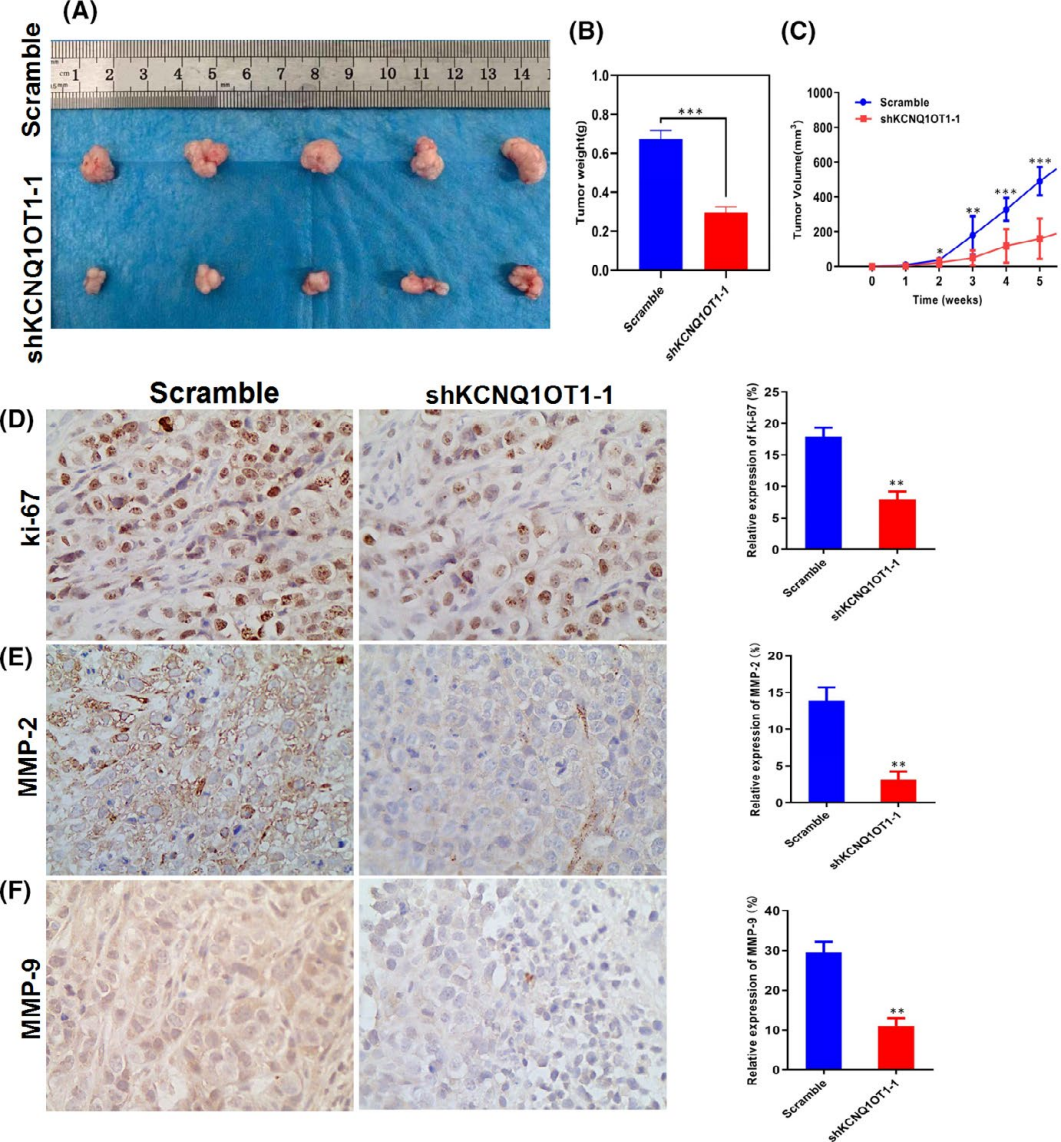
KCNQ1OT1 promotes LSCC growth in vivo. (A‐C) Knockdown of KCNQ1OT1 impaired the growth of xenograft tumours. (D) Representative images of IHC staining of Ki‐67 in xenograft tumour tissues under ×400 magnification. (E‐F) Representative images of IHC staining of MMP‐2 and MMP‐9 in xenograft tumour tissues

Furthermore, the KCNQ1OT1 knockdown group and the negative control group were implanted subcutaneously of nude mice, respectively, to construct the xenograft tumour model. Compared with the negative control group, the tumour volume and weight of KCNQ1OT1 knockdown group were significantly reduced (Figure 4A–C). Ki‐67 expression in vivo is one of the key indicators of cell growth. The results of IHC showed that the expression of Ki‐67 was significantly decreased in KCNQ1OT1 knockdown group (Figure 4D). Matrix Metalloproteinase 2 (MMP‐2) and Matrix Metalloproteinase 9 (MMP‐9) indicate the ability of invasion and metastasis. The results of IHC showed that the immunohistochemical signals of MMP‐2 and MMP‐9 in KCNQ1OT1 knockdown group were much lower than those in negative control group (Figure 4E,F). Overall, KCNQ1OT1 promotes the growth and metastasis of LSCC in vivo.

### 3.5 The expression of ALKBH5 was positively correlated with that of KCNQ1OT1 in LSCC

**FIGURE 5:**
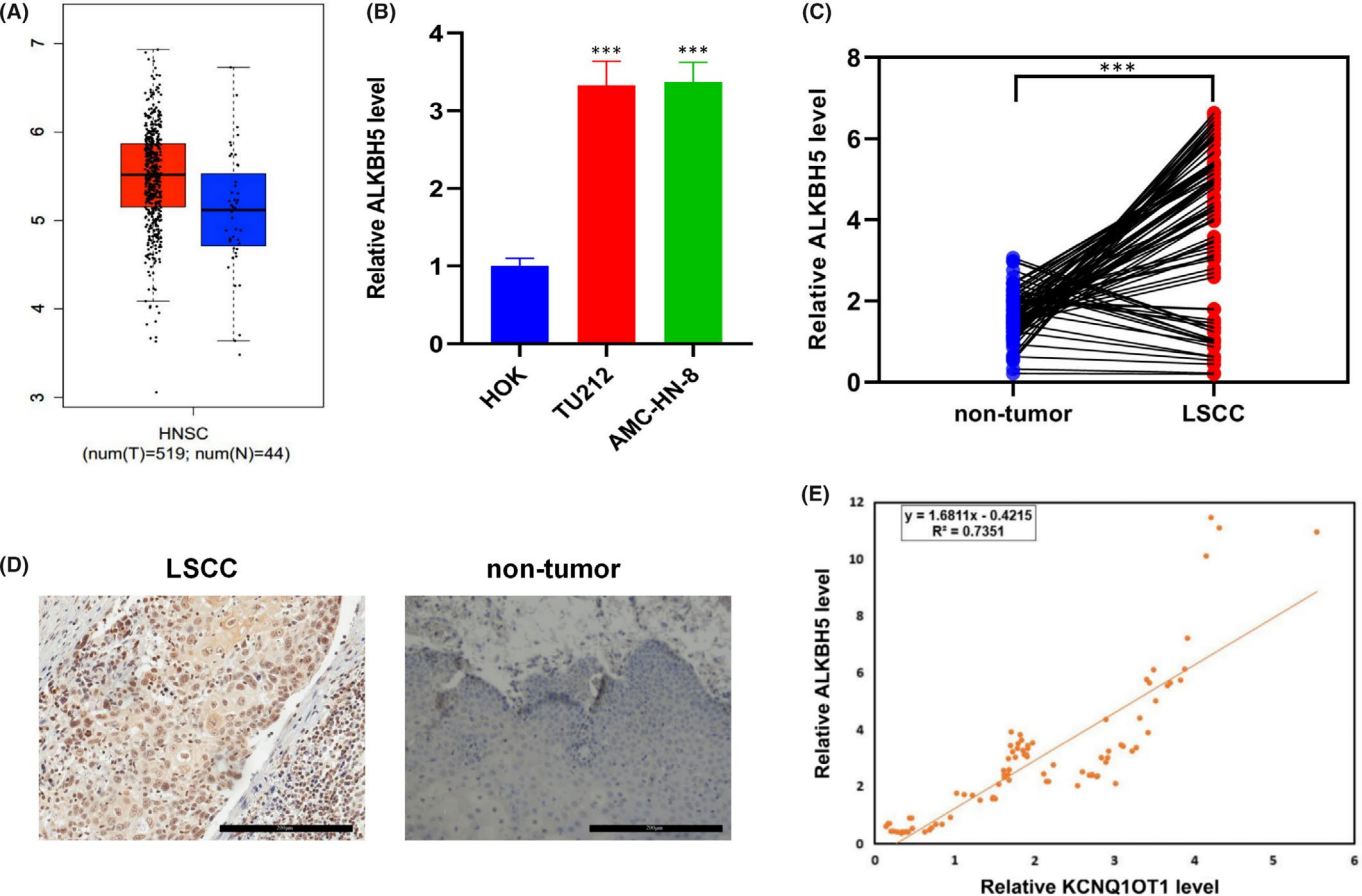
Expression level of ALKBH5 was positively correlated with that of KCNQ1OT1 in LSCC. (A) The expression of ALKBH5 in HNSCC and normal tissues queried from GEPIA database. (B) The mRNA levels of ALKBH5 in HOK cells and LSCC cells was detected by qRT‐PCR. (C) The mRNA levels of ALKBH5 in 86 pairs of LSCC tissues and non‐tumour tissues was determined by qRT‐PCR. (D) Representative images of IHC staining for ALKBH5 protein in LSCC tissues and non‐tumour tissues. (E) The expression of ALKBH5 was significantly associated with the KCNQ1OT1 level in 86 pairs of LSCC

The data from GEPIA showed that the expression level of ALKBH5 in HNSCC is higher than in normal tissues (Figure 5A). Using HOK cells as negative control, the expression of ALKBH5 in TU212 and AMC‐HN‐8 cells was significantly higher than that in HOK cells (Figure 5B). Then, the expression of ALKBH5 in 86 pairs of LSCC and non‐tumour tissues was detected by qRT‐PCR. The results showed that ALKBH5 was highly expressed in LSCC (Figure 5C). The protein expression levels of ALKBH5 in 86 paired LSCC and non‐tumour tissues were detected by IHC. As shown in Figure 5D, ALKBH5 exhibited nuclear localization, and high level of ALKBH5 is observed in LSCC compared with non‐tumour tissues. These results suggested that ALKBH5 was overexpressed in LSCC. Notably, we also found ALKBH5 expression was positively correlated with the KCNQ1OT1 expression in 86 pairs of LSCC (Figure 5E).

### 3.6 ALKBH5 mediates expression of KCNQ1OT1 in an m6A‐YTHDF2‐dependent manner

**FIGURE 6:**
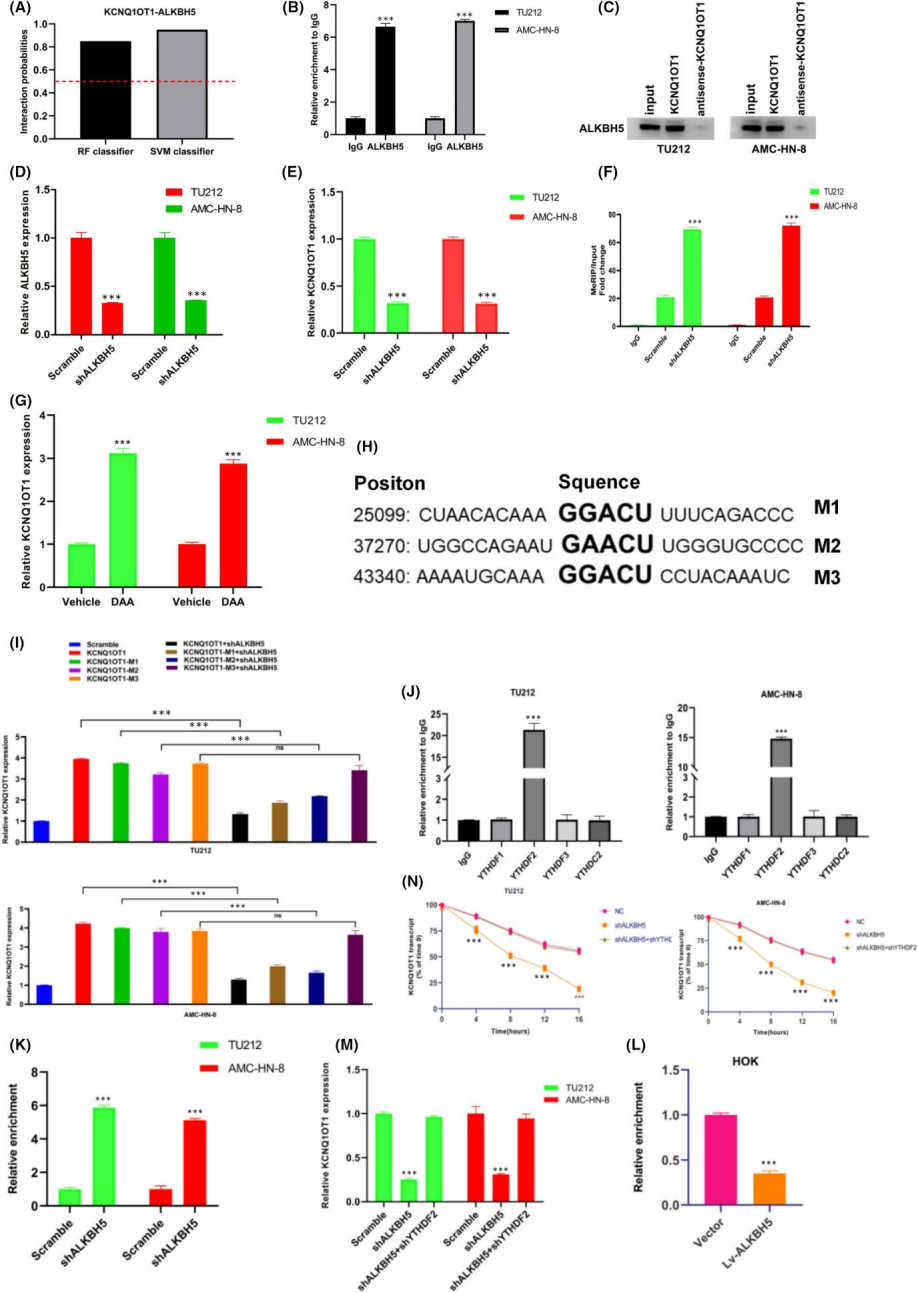
ALKBH5 mediates expression of KCNQ1OT1 in an m6A‐YTHDF2‐dependent manner. (A) The result of prediction of the combination of KCNQ1OT1 and ALKBH5 by using the online software RIPseq. In the performance evaluation experiment, the prediction of probability greater than 0.5 is considered to be ‘positive’. RIP assays and RNA pull‐down analysis were, respectively, used to confirmed the interaction between KCNQ1OT1 and ALKBH5 in (B) and (C). (D) The efficiency of using ALKBH5 lentiviral vector to knockdown the expression of ALKBH5 in TU212 and AMC‐HN‐8 cell lines was verified by qRT‐PCR. (E) qRT‐PCR demonstrated that with the knockdown of ALKBH5, the expression level of KCNQ1OT1 in TU212 and AMC‐HN‐8 cells decreased significantly. (F) MeRIP‐qPCR showed that ALKBH5 knockdown clearly increased the m6A level of KCNQ1OT1. (G) The expression level of KCNQ1OT1 increased after using DDA was detected by qRT‐PCR. (H) The schematic diagram of prediction of three m6A recognition sites in KCNQ1OT1 sequence according to SRAMP. (I) The binding site of m6A was mainly located in M3 region of KCNQ1OT1, which was explained by PCR. (J) The quantitation of RIP assay illustrated m6A ‘reader’ proteins YTHDF2 was apparently connected with KCNQ1OT1. Moreover, the binding of KCNQ1OT1 and YTHDF2 was tighter when ALKBH5 silenced in (K). Oppositely, the interaction was inhibited while ALKBH5 overexpressed in (L). (M) qRT‐PCR was carried out to detect the expression level of KCNQ1OT1 with the knockdown of YTHDF2 and ALKBH5 separately or simultaneously. (N) The half‐life curve exhibited that the deletion of YTHDF2 rescued the change of the half‐life of KCNQ1OT1 transcript caused by ALKBH5 knockdown

We speculated whether the low m6A methylation of KCNQ1OT1 in LSCC is due to the binding with ALKBH5. The online software RIPseq was used to predict the combination of KCNQ1OT1 and ALKBH5. The results showed that random forest (RF) classifier was 0.85 and support vector machine (SVM) classifier was 0.95 (Figure 6A), which means that the corresponding RNA and protein are likely to interact. The interaction between KCNQ1OT1 and ALKBH5 was further verified by RIP assays and RNA pull‐down analysis (Figure 6B,C). TU212 and AMC‐HN‐8 cells were transfected with ALKBH5 lentiviral vector. (Figure 6D). This raised the question whether ALKBH5 regulates KCNQ1OT1 expression through m6A methylation‐dependent manner. To test this hypothesis, we first knocked down ALKBH5 in TU212 and AMC‐HN‐8 cells resulted in decreased expression of KCNQ1OT1 (Figure 6E). MeRIP‐qPCR was used to confirm that ALKBH5‐mediated m6A demethylation of KCNQ1OT1. As expected, ALKBH5 knockdown significantly increased the m6A level of KCNQ1OT1, which confirmed that KCNQ1OT1 transcripts were functionally important substrates of ALKBH5 (Figure 6F). Treatment with a global methylation inhibitor (DAA) led to the upregulation of KCNQ1OT1 levels in LSCC cells (Figure 6G). Then, we predicted three m6A recognition sites with very high confidence in KCNQ1OT1 sequence based on SRAMP (Figure 6H). Then, three m6A recognition sites of KCNQ1OT1 were mutated (GAC to GCC). When ALKBH5 was knockdown, KCNQ1OT1‐M1 and KCNQ1OT1‐M2 decreased significantly, indicating that m6A mainly regulates KCNQ1OT1 expression in LSCC cells through M3 region, although M1 and M2 may also be recognized to some extent (Figure 6I). The results showed that most of m6A were enriched in M3 region of KCNQ1OT1.

The next question we needed to investigate was how m6A affects KCNQ1OT1 expression. Published studies have shown that m6A ‘reader’ proteins selectively recognize and mediate the degradation of m6A‐containing RNA.^23^ In order to detect which reader protein is responsible for ALKBH5‐mediated KCNQ1OT1 upregulation, we used YTHDF1, YTHDF2, YTHDF3 and YTHDC2 antibodies to carry out RIP analysis. The results showed that only YTHDF2 was significantly associate with KCNQ1OT1 (Figure 6J). Furthermore, the interaction between KCNQ1OT1 and YTHDF2 was enhanced after silencing ALKBH5 expression in TU212 and AMC‐HN‐8 cells (Figure 6K), while ALKBH5 overexpression in HOK cells inhibited this interaction (Figure 6L). In order to detect whether YTHDF2 is involved in ALKBH5‐mediated KCNQ1OT1 upregulation, we further knockdown YTHDF2 in LSCC cells to detect the change of KCNQ1OT1 expression. The results had identified that knockdown of ALKBH5 decreased the expression of KCNQ1OT1 and increased the m6A methylation level. YTHDF2 and ALKBH5 knockdown at the same time rescued the decrease of KCNQ1OT1 expression (Figure 6M). We further investigated whether YTHDF2 regulates KCNQ1OT1 expression by regulating its stability. We used the transcription inhibitor actinomycin D to detect the half‐life of KCNQ1OT1 transcripts in each group, respectively. Indeed, the half‐life of KCNQ1OT1 in both cells with ALKBH5 knockdown was reversed after deletion of YTHDF2 expression (Figure 6N). Therefore, ALKBH5 mediates KCNQ1OT1 expression via an m6A‐YTHDF2‐dependent manner.

### 3.7 KCNQ1OT1 directly interacts with HOXA9 in LSCC

**FIGURE 7:**
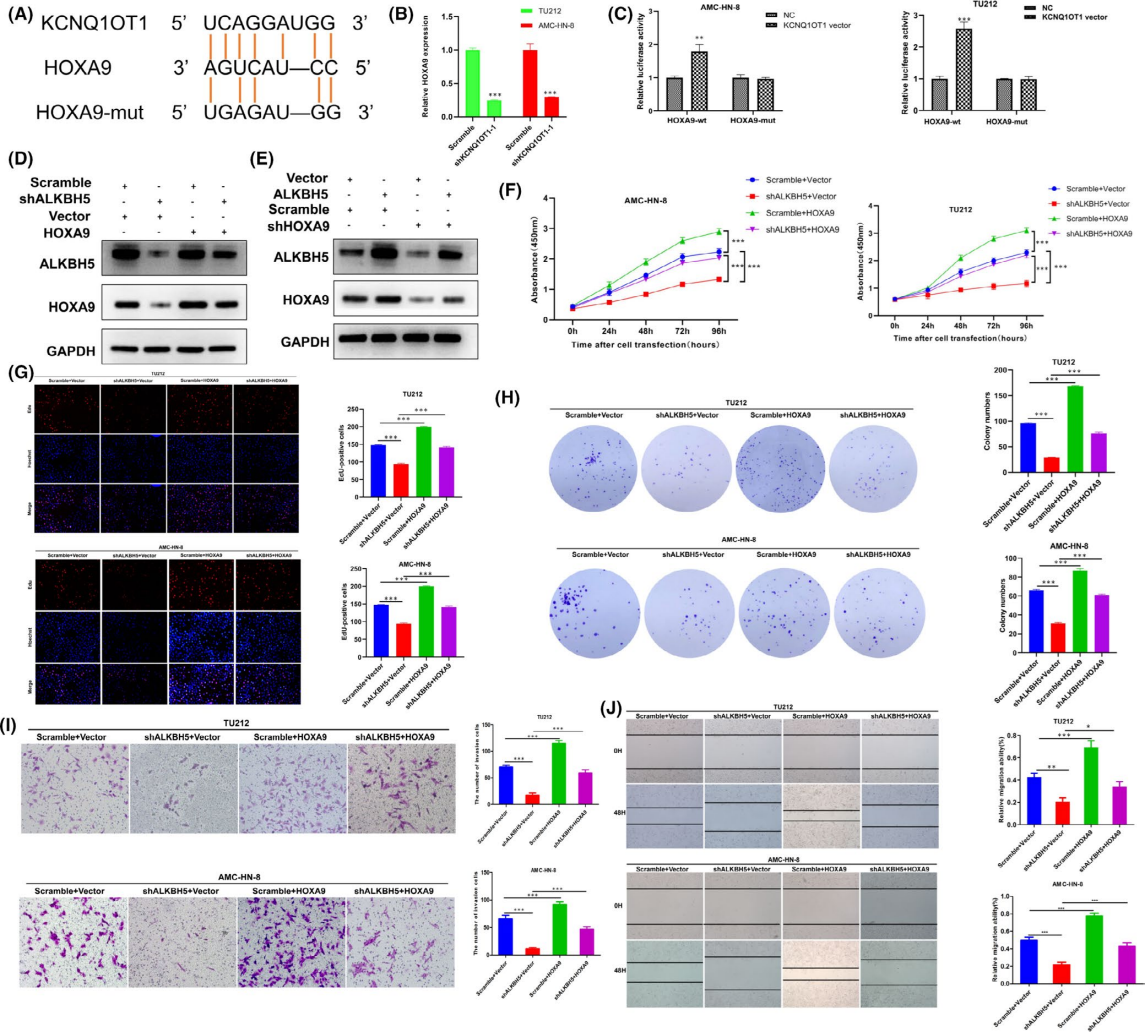
KCNQ1OT1 directly interacts with HOXA9 in LSCC. (A) Schematic diagram of potential binding sites between KCNQ1OT1 and HOXA9 predicted by Starbase 3.0. (B) The expression level of HOXA9 in LSCC cells was significantly reduced after KCNQ1OT1 was knockdown was detected by qRT‐PCR. (C) HOXA9 was confirmed as a target of KCNQ1OT1 by luciferase reporter activity assay after transfection of wild‐type vector and mutant vector. (D‐E) The protein expression level of ALKBH5 and HOXA9 was determined by Western blot. (F‐J) CCK‐8, Edu, colony formation, transwell and wound healing assays confirmed that the ectopic expression of HOXA9 promoted the progress of LSCC, and partially rescued the decline of LSCC cell proliferation and invasion after ALKBH5 knockdown

Starbase 3.0 was used to predict mRNA as a target for KCNQ1OT1. The results showed that there was a direct binding site between KCNQ1OT1 and HOXA9. The potential complementary binding sites of HOXA9 and KCNQ1OT1 were shown in Figure 7A. Previous studies have shown that HOXA9 was significantly upregulated in LSCC tissues compared to control tissues.^24^, ^25^ The analysis of TCGA database showed that HOXA9 was highly expressed in HNSCC, and its expression level was related to tumour grade and lymph node metastasis status (Figure S3A–C). qRT‐PCR verified that HOXA9 was highly expressed in LSCC tissues compared with non‐tumour tissues in 86 pairs of LSCC sample cohort, and its expression was positively correlated with the expression level of KCNQ1OT1 (Figure S4A,B). Besides, we found that high expression of HOXA9 was significantly associated with poor prognosis (Figure S4C). Using HOK cells as a negative control group, it was found that the expression of HOXA9 in TU212 and AMC‐HN‐8 was significantly higher (Figure S4D). IHC showed HOXA9 was expressed significantly higher in LSCC tissues and mainly located in the nucleus (Figure S5). Next, we changed the expression of HOXA9 in TU212 and AMC‐HN‐8 (Figure S6A,B). CCK‐8, Edu, colony formation, transwell and wound healing assays demonstrated that the silencing of HOXA9‐inhibited LSCC cells proliferation, migration and invasion ability (Figure S7A–E). The results of qRT‐PCR showed that the expression of HOXA9 decreased significantly after KCNQ1OT1 knockdown (Figure 7B). Luciferase reporter assays were used to further verify that HOXA9 was the target of KCNQ1OT1. The results showed that luciferase activity increased after transfection with wild‐type vector. However, the luciferase activity of the mutant type vector was not affected (Figure 7C). Culturing KCNQ1OT1 knockdown LSCC cell line TU212 and AMC‐HN‐8 with medium containing the transcription inhibitor actinomycin D to block mRNA synthesis, and the results indicated that HOXA9 mRNA attenuation rate was faster in KCNQ1OT1 knockdown LSCC cells (Figure S8). The inhibition of ALKBH5 could partially neutralize the effect of overexpressed HOXA9 on HOXA9 protein expression in TU212 cells (Figure 7D), while ALKBH5 overexpression could partially neutralize the effect of HOXA9 inhibition on the expression of HOXA9 protein in AMC‐HN‐8 cells (Figure 7E). In addition, CCK‐8, Edu, colony formation, transwell and wound healing assays confirmed that the ectopic expression of HOXA9 promoted the progress of LSCC, and partially rescued the decline of LSCC cell proliferation and invasion after ALKBH5 knockdown (Figure 7F‐J).

## 4 DISCUSSION

The classification of head and neck tumours includes tumours of nasal cavities, paranasal sinuses, oral cavity and larynx, nasopharynx, oropharynx and hypopharynx.^26^ More than 90% of head and neck tumours are squamous cell carcinoma^27^; therefore, they are classified as HNSCC.^28^ LSCC is one of the largest subtypes.^26^, ^27^ Increasing evidences show that lncRNAs play an important role in the development of tumour and have been proved to be important regulators of signal pathways in the carcinogenesis.^29^ m6A is a prevalent internal modification of mRNAs, which regulates gene expression by modulating RNA processing, localization and translation. In this study, we used the Epitranscriptomic Microarray to detect the differential expression of m6A‐lncRNA in three pairs of LSCC and non‐tumour tissues. Combining MeRIP‐qPCR experiment, we verified that only KCNQ1OT1 showed significantly low m6A methylation in LSCC samples. KCNQ1OT1 is located at the human KCNQ1 locus, with a length of 91 kb. Recent study confirms that KCNQ1OT1 plays essential roles in tumour proliferation, metabolism, epithelial‐mesenchymal transition (EMT) and growth by acting as competing endogenous RNA (ceRNA).^30^, ^31^ It has been proved that KCNQ1OT1 can promote cell proliferation and autophagy and inhibit cell apoptosis by regulating mir‐204‐5p/ATG3 axis, which provides a promising target for non‐small cell lung cancer (NSCLC) treatment.^32^ Our investigation found that KCNQ1OT1 shows low m6A methylation and high expression in LSCC tissues. Based on clinical data, in vivo and in vitro experiments, it is confirmed that the expression level of KCNQ1OT1 is closely related to the development and prognosis of LSCC.

In mammals, m6A modification occurs through methyltransferase complex, which is mainly composed of METTL3, METTL14, WTAP and other components. This modification can be reversed by demethylase FTO and ALKBH5.^33^, ^34^ Chao et al. demonstrate that ALKBH5 affects the proliferation and invasion of lung adenocarcinoma cells under IH by downregulating m6A modification on FOXM1 mRNA and by promoting FOXM1 expression.^35^ Other studies have revealed a new mechanism by which ALKBH5 methylation lncRNA KCNK15‐AS1 inhibits the movement of pancreatic cancer.^36^ Our results suggest that ALKBH5 is overexpression and positively correlated with that of KCNQ1OT1 in LSCC. ALKBH5 affects the m6A methylation level of KCNQ1OT1 by regulating specific sites on KCNQ1OT1. Mechanistically, ALKBH5 reduced the m6A methylation level of KCNQ1OT1, and subsequently decreases the binding level of KCNQ1OT1 in reader protein YTHDF2. It is further confirmed that there is a direct binding site between KCNQ1OT1 and HOXA9, and rescue experiments demonstrate that ALKBH5‐KCNQ1OT1‐HOXA9 axis affects tumour proliferation, invasion and metastasis. This means that ALKBH5‐KCNQ1OT1‐HOXA9 axis may be an effective target for the diagnosis and treatment of LSCC.

For the first time, we integrally analysed differential expression of m6A‐lncRNA in LSCC and non‐tumour tissues, revealing a tight relationship between m6A methylation and the emergence of LSCC. We found ALKBH5 increase can promote the development of LSCC by promoting KCNQ1OT1‐HOXA9 signalling via a m6A‐YTHDF2‐dependent manner. However, whether the combined expression of ALKBH5‐KCNQ1OT1‐HOXA9 can be used as a diagnosis and treatment biomarker of LSCC needs more sample data. This study enriched the research on the pathogenesis of LSCC and provided a new idea for the diagnosis and treatment of LSCC. In future, our team will continue to explore the potential clinical significance of lncRNA methylation and its methylation modification in LSCC.

## CONFLICT OF INTEREST

The authors declare that they have no competing interests.

## AUTHOR CONTRIBUTIONS

**Yushan Li:** Conceptualization (equal); Data curation (equal); Formal analysis (equal); Investigation (equal); Methodology (equal); Validation (equal); Visualization (equal); Writing – original draft (equal). **Bingrui Yan:** Data curation (equal); Formal analysis (equal); Investigation (equal); Methodology (equal). **Xin Wang:** Validation (equal); Writing – review & editing (equal). **Qiuying Li:** Formal analysis (equal); Investigation (equal). **Xuan Kan:** Investigation (equal); Methodology (equal). **Jingting Wang:** Data curation (equal); Validation (equal). **Ya‐nan Sun:** Conceptualization (equal); Funding acquisition (equal); Project administration (equal); Resources (equal); Supervision (equal); Writing – review & editing (equal). **Peng Wang:** Conceptualization (equal); Data curation (equal); Formal analysis (equal); Funding acquisition (equal); Methodology (equal). **Linli Tian:** Data curation (equal); Investigation (equal); Supervision (equal). **Ming Liu:** Supervision (equal); Writing – review & editing (equal).

## Supporting information

Fig S1

Fig S2

Fig S3

Fig S4

Fig S5

Fig S6

Fig S7

Fig S8

Table S1

Table S2

## Data Availability

The authors declare that the data in this article are available.
