# Correction to “ALKBH5‐Mediated m6A Modification of lncRNA KCNQ1OT1 Triggers the Development of LSCC via Upregulation of HOXA9”

**DOI:** 10.1111/jcmm.71091

**Published:** 2026-04-11

In Figure 3D, Figure 7H,

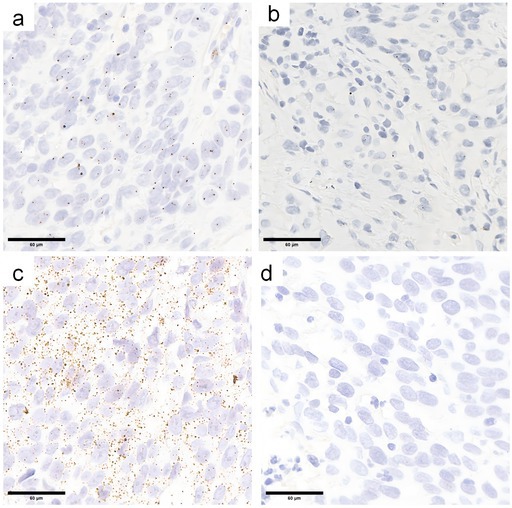

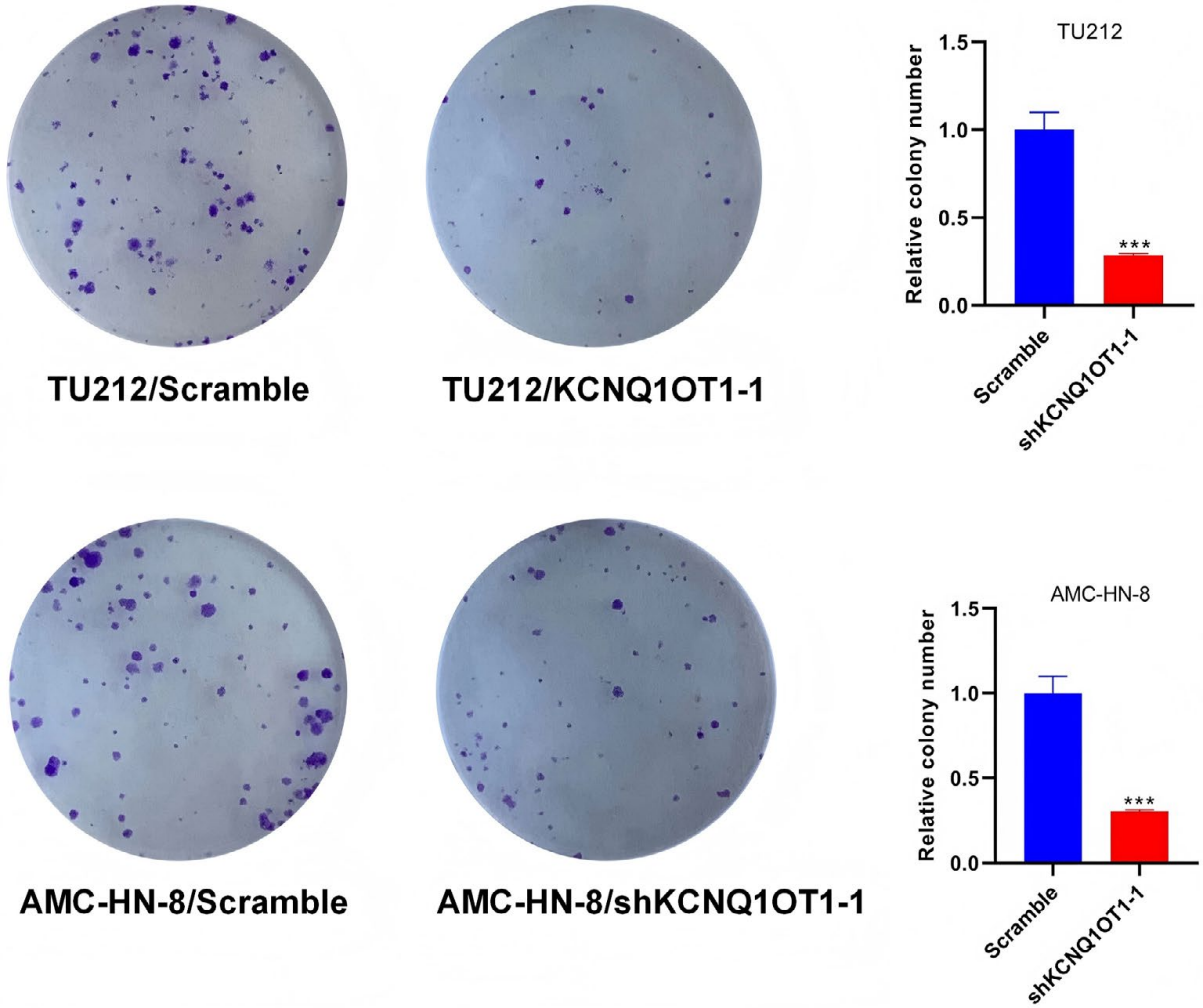

Cell Colony Assay images were mistakenly selected from a control group folder and in Figure 2F.

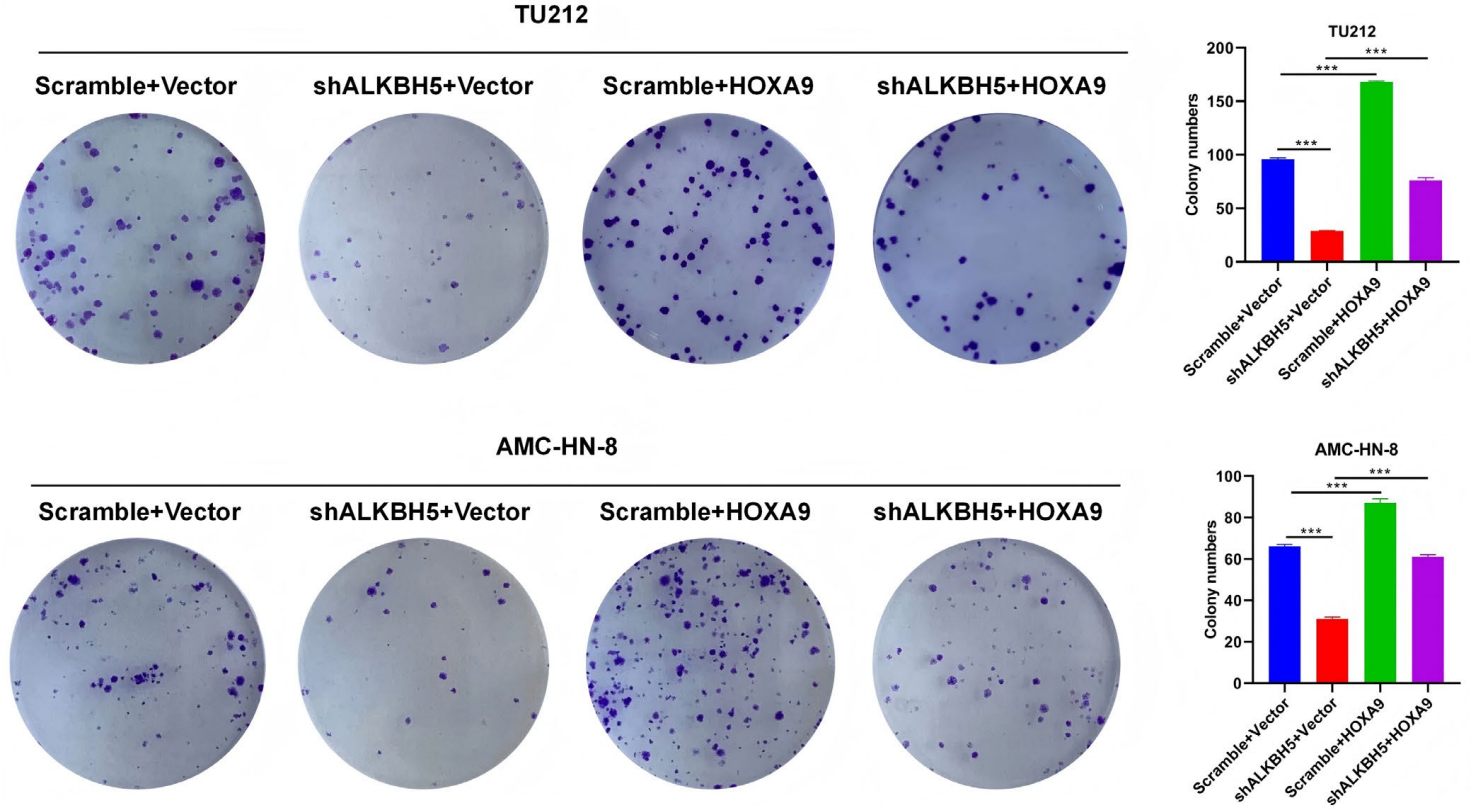

Image labeled “paracancerous tissue” was incorrectly taken from a different patient cohort's adjacent normal tissue folder; correct image is from the matching paracancerous tissue of the same cancer patient.

The aforementioned image errors resulted from accidental confusion of experimental data folders during final manuscript preparation (no data fabrication, manipulation, or academic misconduct). The corrected images are consistent with the original experimental records and experimental design. These corrections do not affect the study's core conclusions, experimental validity, or analytical framework: (1) For the cell colony assay, the quantitative colony formation rate data remains accurate and consistent with the correct original images; (2) For the ISH assay, the quantitative results of hybridization signals in tumor vs. paracancerous tissues and statistical comparisons between groups remain unchanged. The correct paracancerous image only fixes sample matching without altering the observed biological trend or conclusion.

The corrected Figure 2F, Figure 3D, Figure 7H are shown below.

We apologize for these errors.

