# Supplementary material for: ALKBH5‐mediated m6A modification of lncRNA KCNQ1OT1 triggers the development of LSCC via upregulation of HOXA9
Source: J Cell Mol Med. 2021 Dec 1;26(2):385–98. doi: 10.1111/jcmm.17091 (PMC8743647; doi:10.1111/jcmm.17091)
Supplement: Supplementary file 1 — Fig S1 [file JCMM-26-385-s005.doc]

**Figure S1**


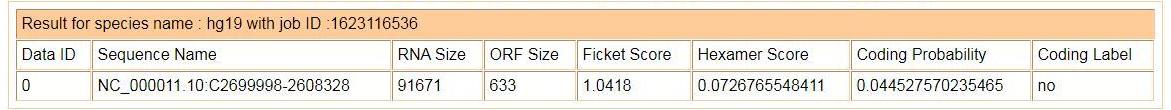


**Figure S1**. The result of prediction of the protein coding potential of KCNQ1OT1 by using the online software RNA coding potential assessment tool (CPAT).
