# Supplementary material for: ALKBH5‐mediated m6A modification of lncRNA KCNQ1OT1 triggers the development of LSCC via upregulation of HOXA9
Source: J Cell Mol Med. 2021 Dec 1;26(2):385–98. doi: 10.1111/jcmm.17091 (PMC8743647; doi:10.1111/jcmm.17091)
Supplement: Supplementary file 2 — Fig S2 [file JCMM-26-385-s007.doc]

**Figure S2**

**
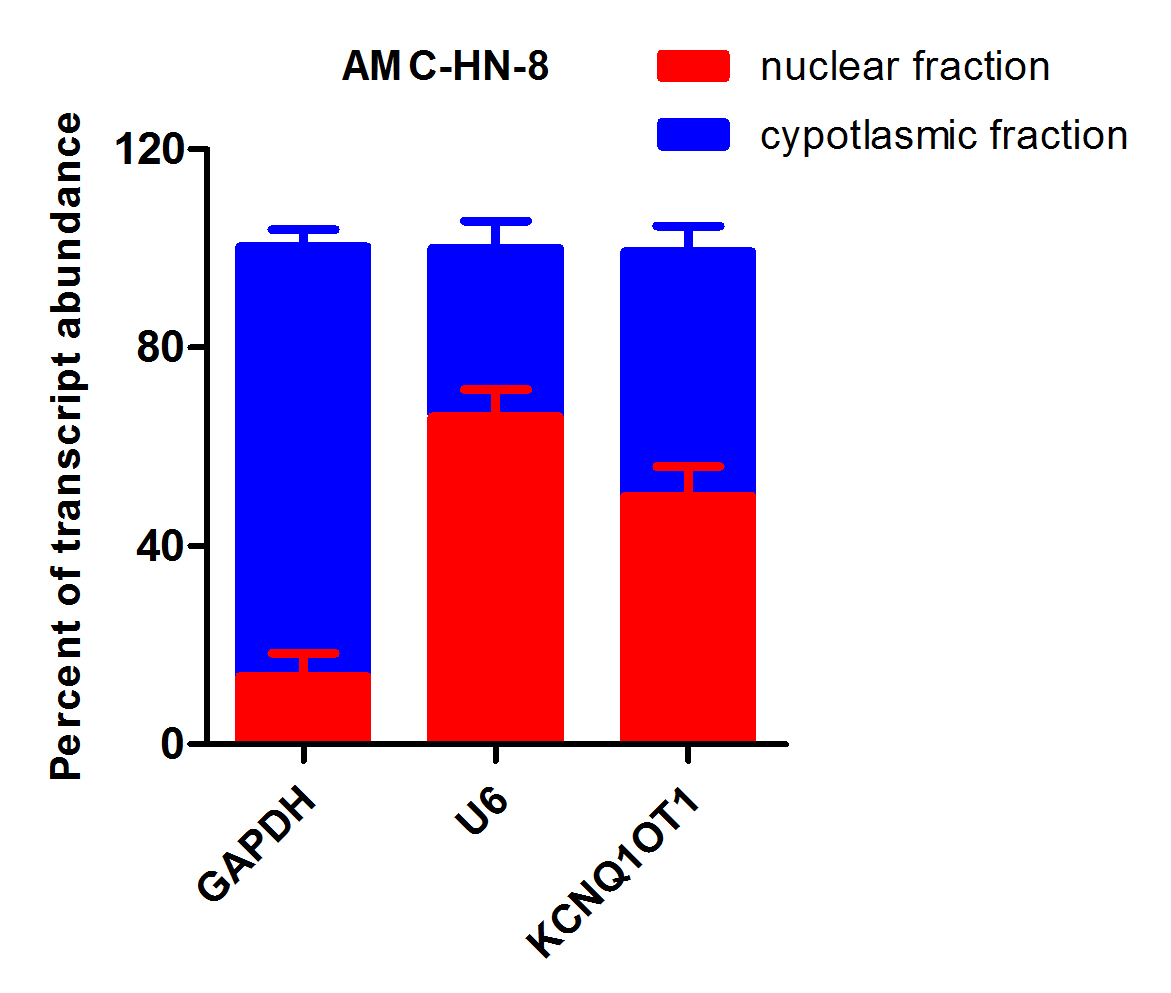
**

**
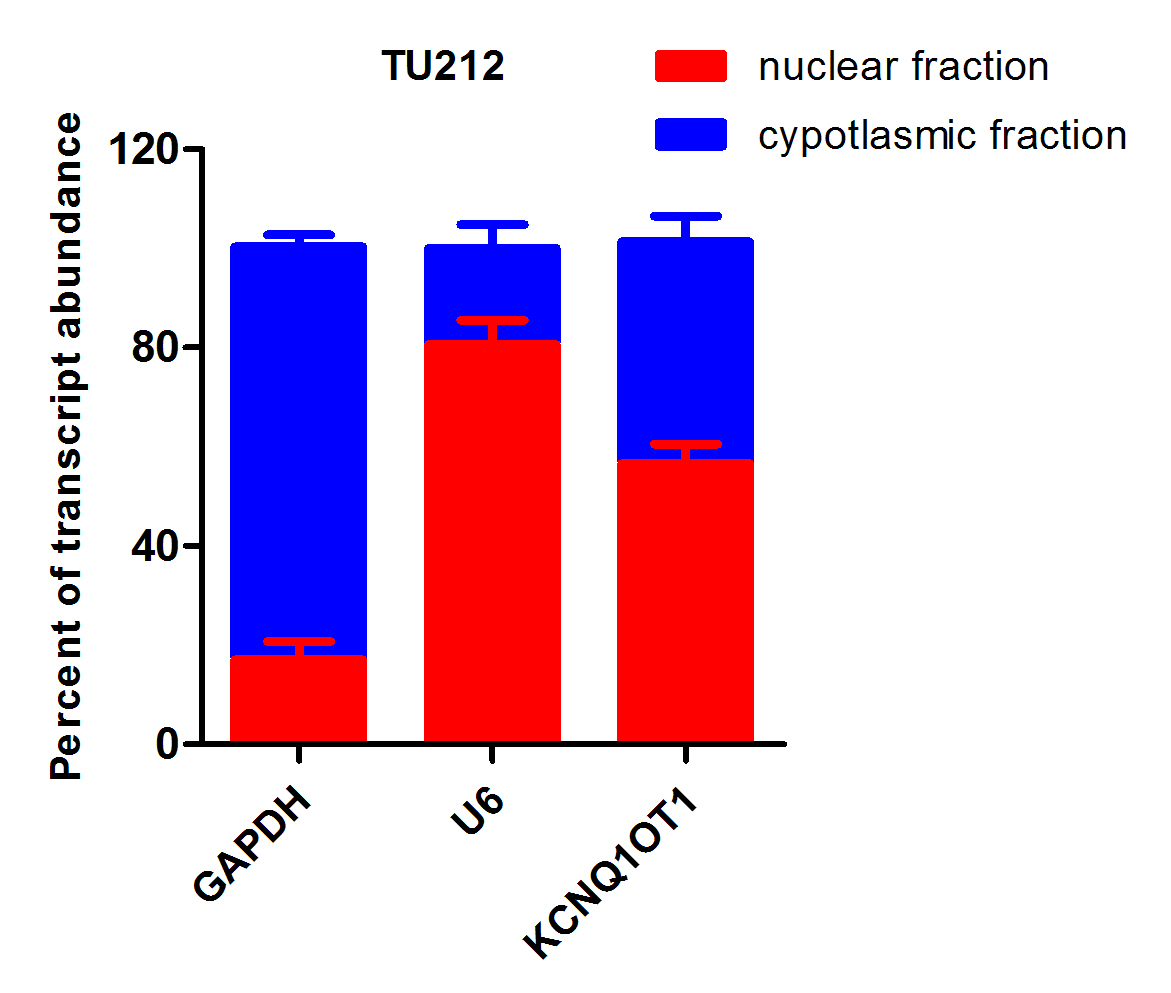
**

**Figure S2**. QRT-PCR was performed to detect the expression level of KCNQ1OT1 in nucleus or cytoplasm of LSCC cell line TU212 and AMC-HN-8. GAPDH was used as a cytoplasmic marker and U6 was used as a nuclear marker.
