# Supplementary material for: ALKBH5‐mediated m6A modification of lncRNA KCNQ1OT1 triggers the development of LSCC via upregulation of HOXA9
Source: J Cell Mol Med. 2021 Dec 1;26(2):385–98. doi: 10.1111/jcmm.17091 (PMC8743647; doi:10.1111/jcmm.17091)
Supplement: Supplementary file 3 — Fig S3 [file JCMM-26-385-s008.doc]

**Figure S3**

**A**

**B**

**C**

**
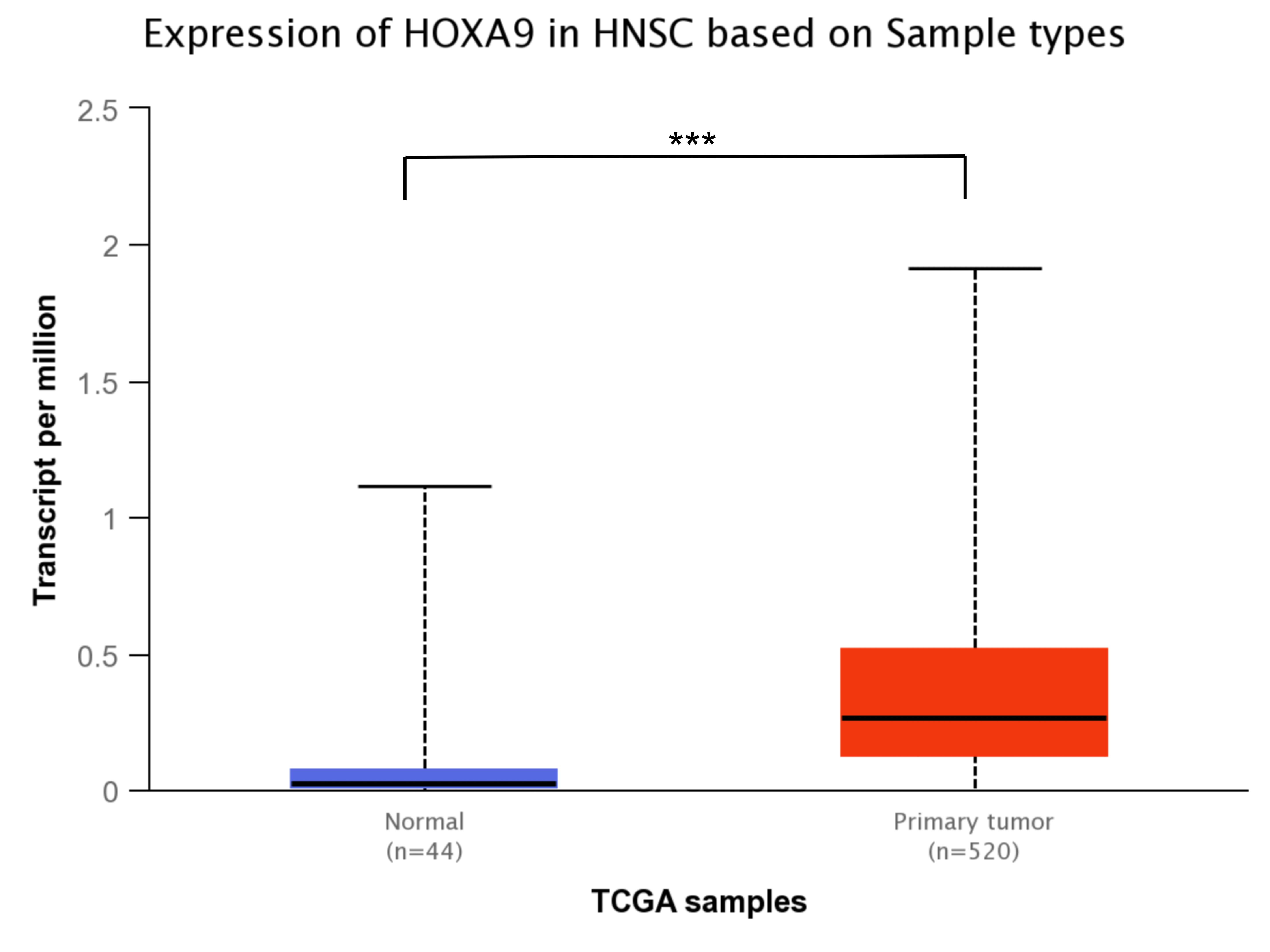

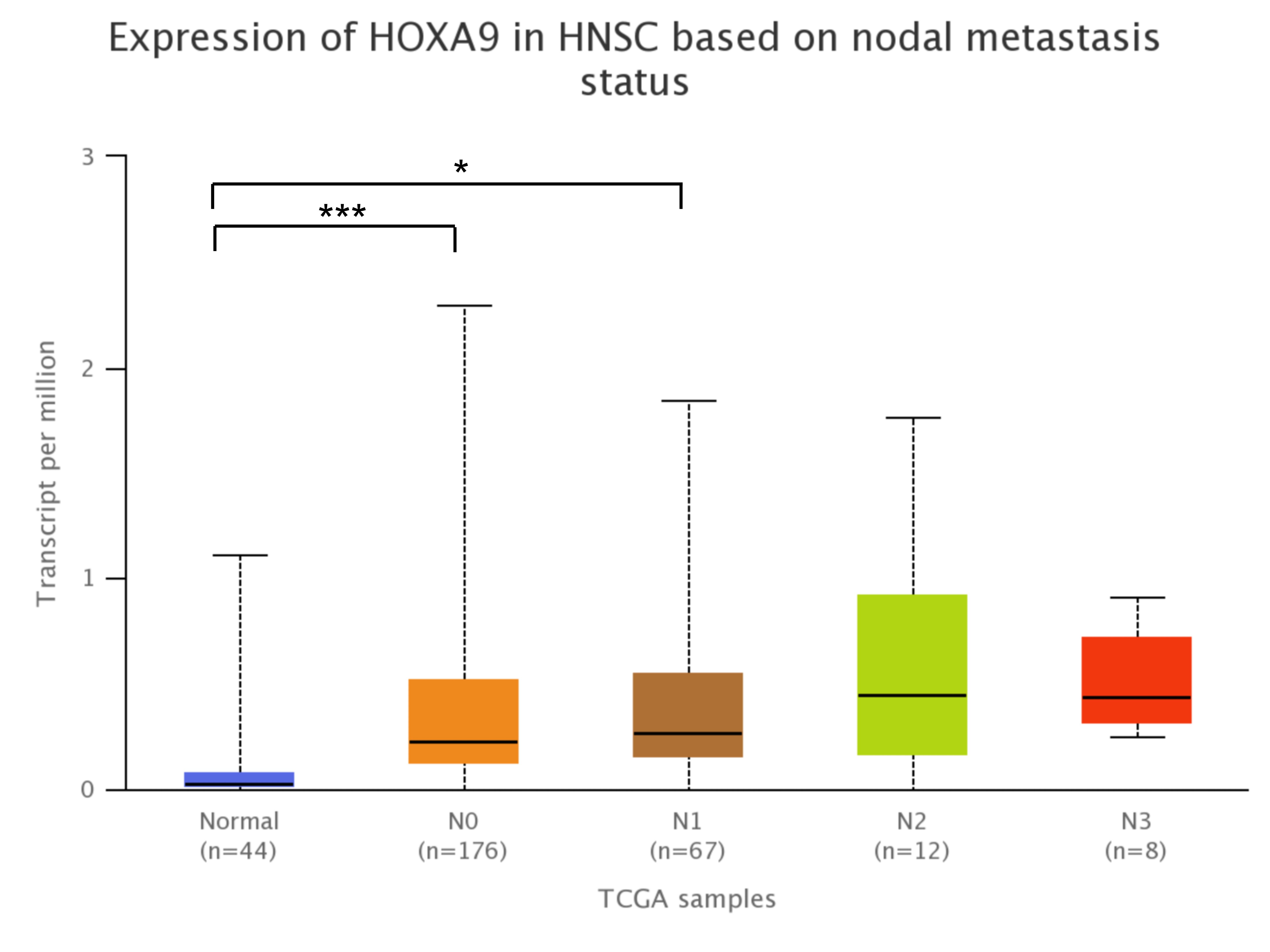

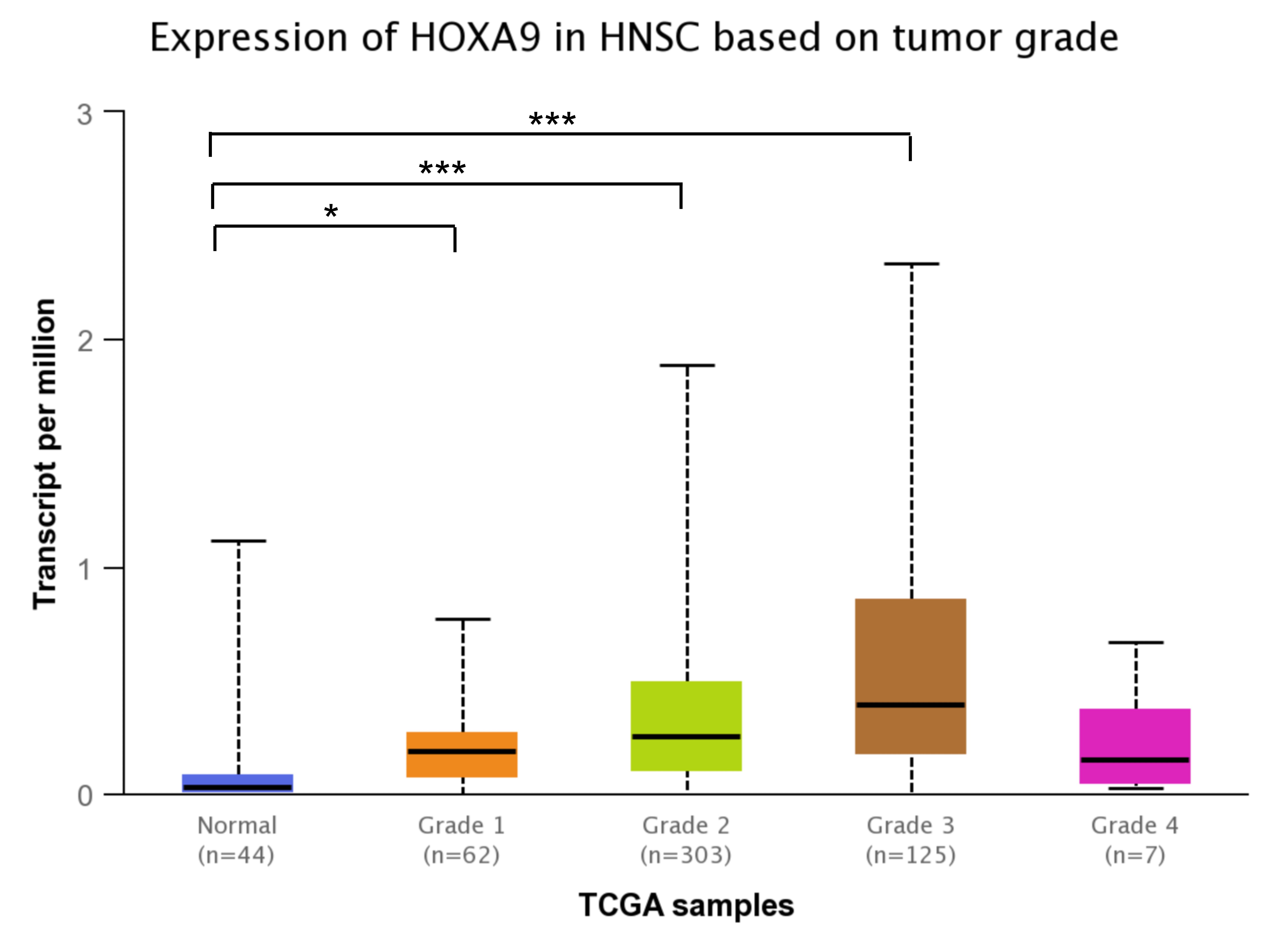
**

**Figure S3**. (A-C) Based on TCGA database, HOXA9 was overexpressed in HNSC and related to tumor grade and lymph node metastasis status.
