# Supplementary material for: ALKBH5‐mediated m6A modification of lncRNA KCNQ1OT1 triggers the development of LSCC via upregulation of HOXA9
Source: J Cell Mol Med. 2021 Dec 1;26(2):385–98. doi: 10.1111/jcmm.17091 (PMC8743647; doi:10.1111/jcmm.17091)
Supplement: Supplementary file 4 — Fig S4 [file JCMM-26-385-s006.doc]

**Figure S4**

**
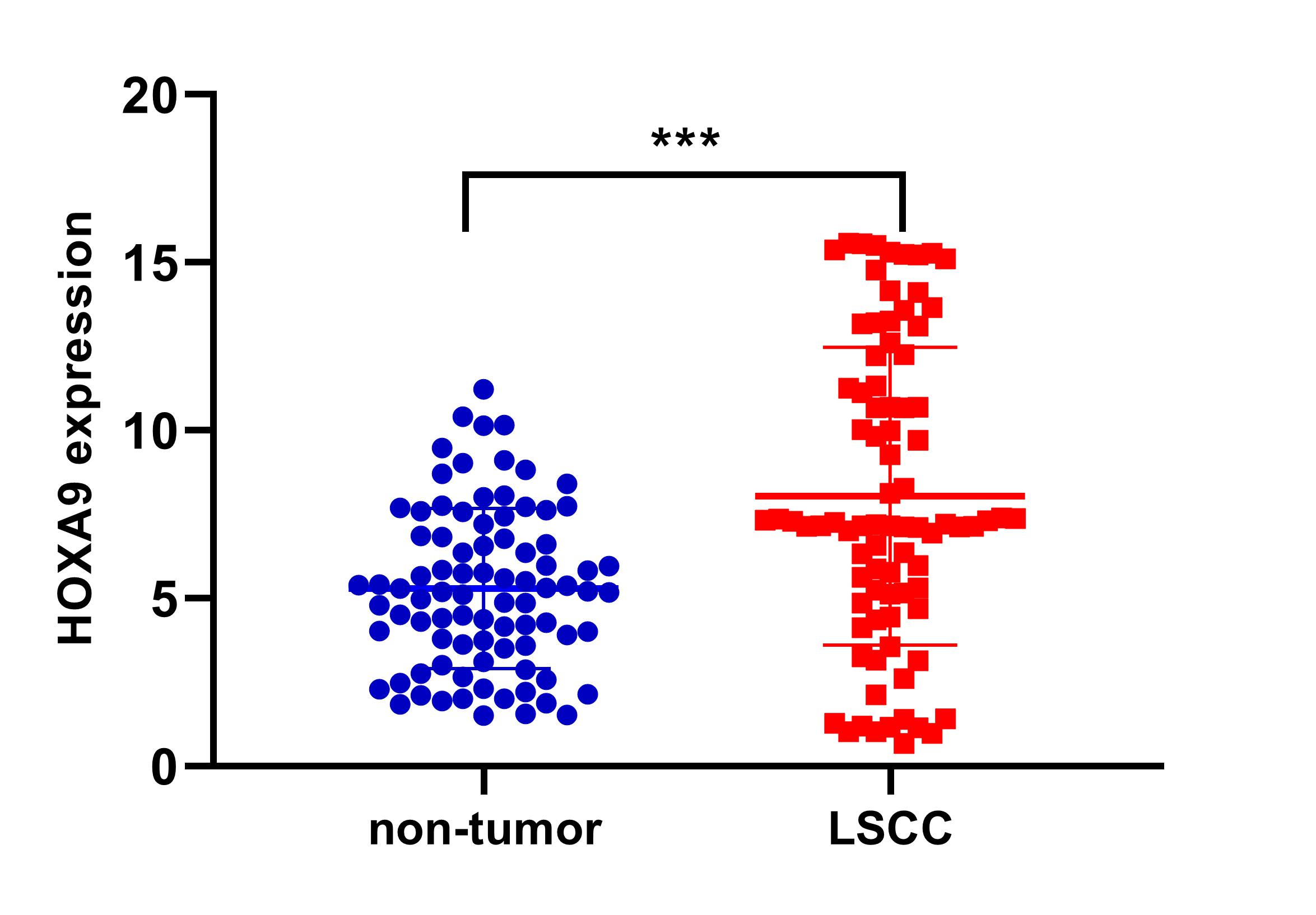

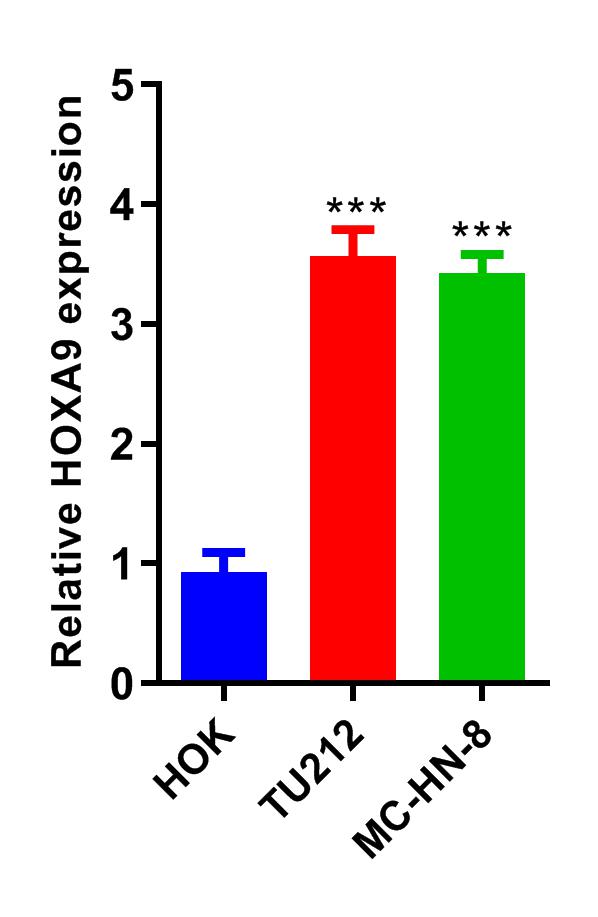
**

**A**

**B**

**C**

**
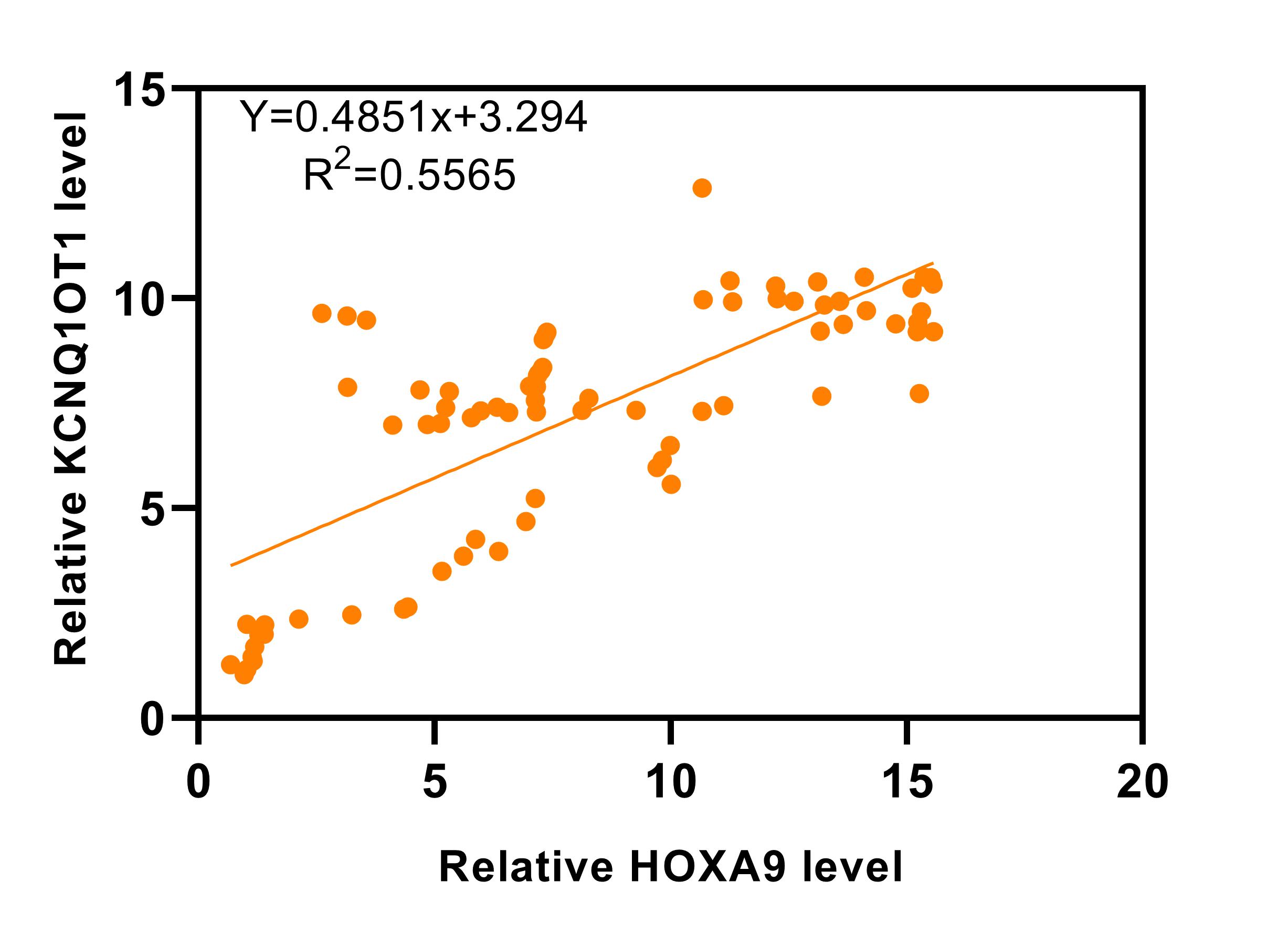
**

**
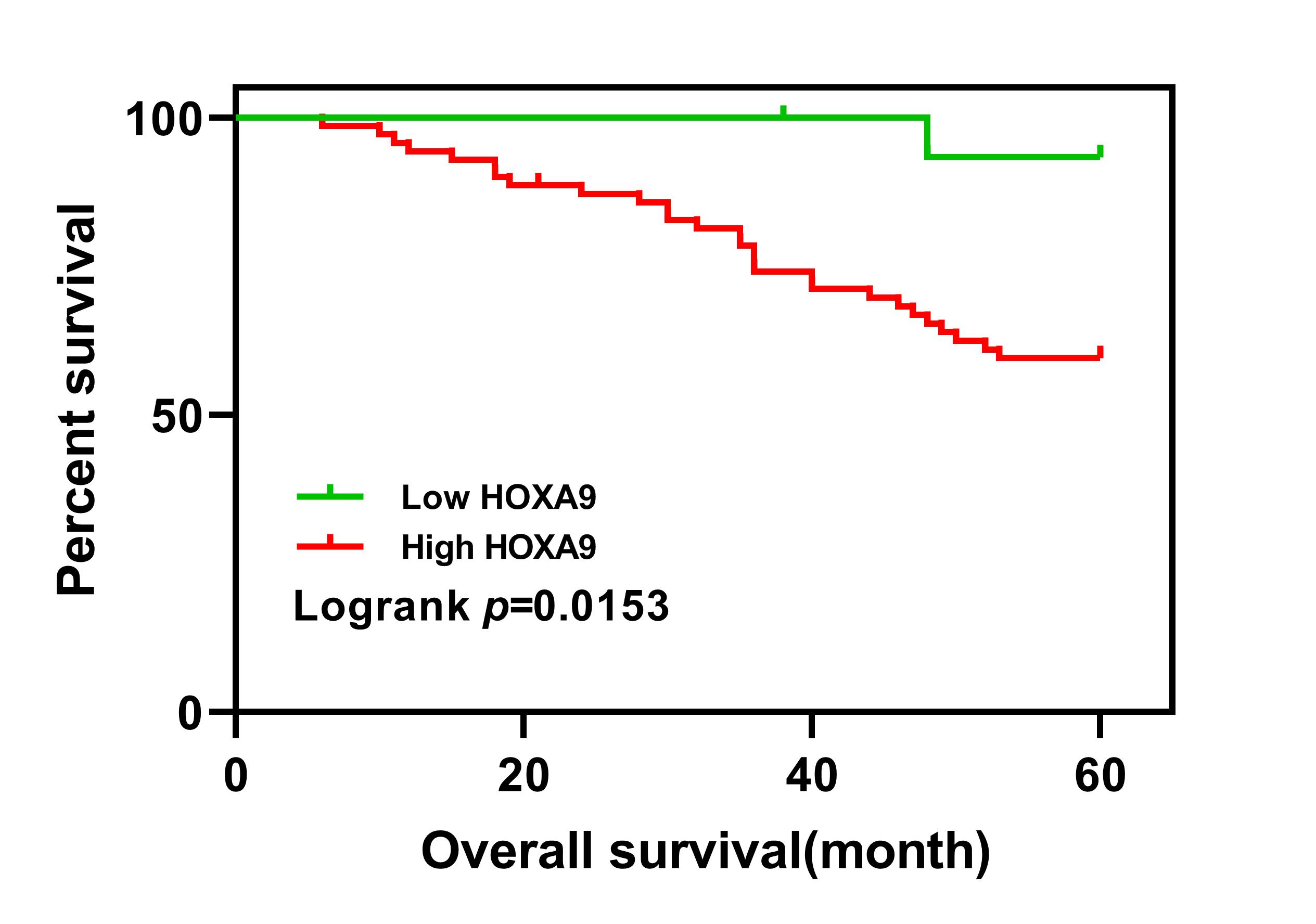
**

**D**

**Figure S4**. (A) QRT-PCR was used to verify the expression of HOXA9 in 86 pairs of LSCC and non-tumor tissues. (B) HOXA9 expression was positively associated with the expression level of KCNQ1OT1 in 86 pairs of LSCC. (C) The mRNA levels of HOXA9 in HOK cells and LSCC cells were detected by qRT-PCR. (D) High expression of HOXA9 was significantly associated with poor prognosis.
