# Supplementary material for: ALKBH5‐mediated m6A modification of lncRNA KCNQ1OT1 triggers the development of LSCC via upregulation of HOXA9
Source: J Cell Mol Med. 2021 Dec 1;26(2):385–98. doi: 10.1111/jcmm.17091 (PMC8743647; doi:10.1111/jcmm.17091)
Supplement: Supplementary file 5 — Fig S5 [file JCMM-26-385-s002.doc]

**Figure S5**

**LSCC**

**Non-tumor**

**
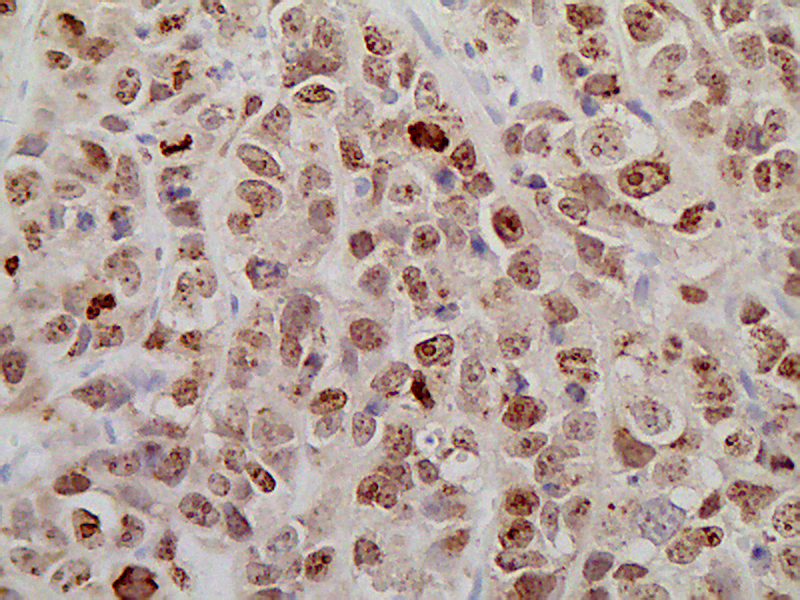

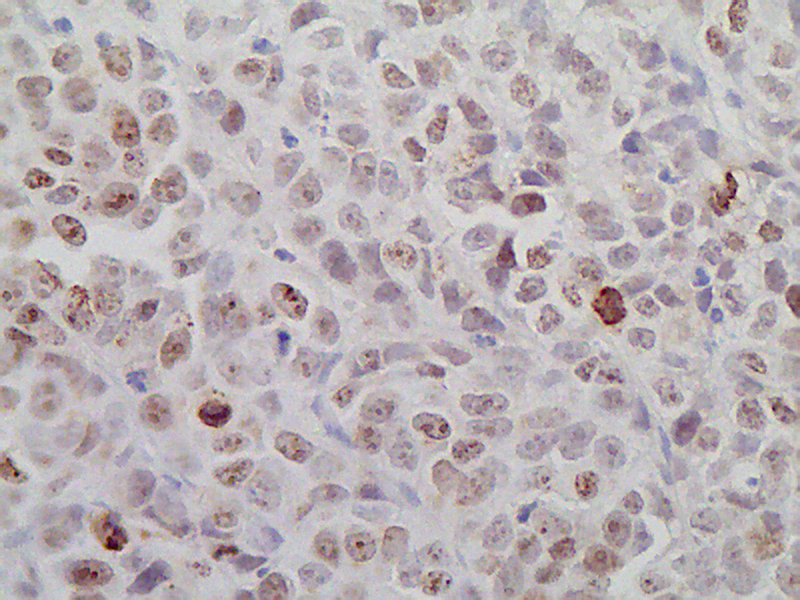
**

**Figure S5**. Representative IHC images of HOXA9 expression in 86 pairs of LSCC and non-tumor tissues under ×400 magnification.
