# Supplementary material for: ALKBH5‐mediated m6A modification of lncRNA KCNQ1OT1 triggers the development of LSCC via upregulation of HOXA9
Source: J Cell Mol Med. 2021 Dec 1;26(2):385–98. doi: 10.1111/jcmm.17091 (PMC8743647; doi:10.1111/jcmm.17091)
Supplement: Supplementary file 6 — Fig S6 [file JCMM-26-385-s009.doc]

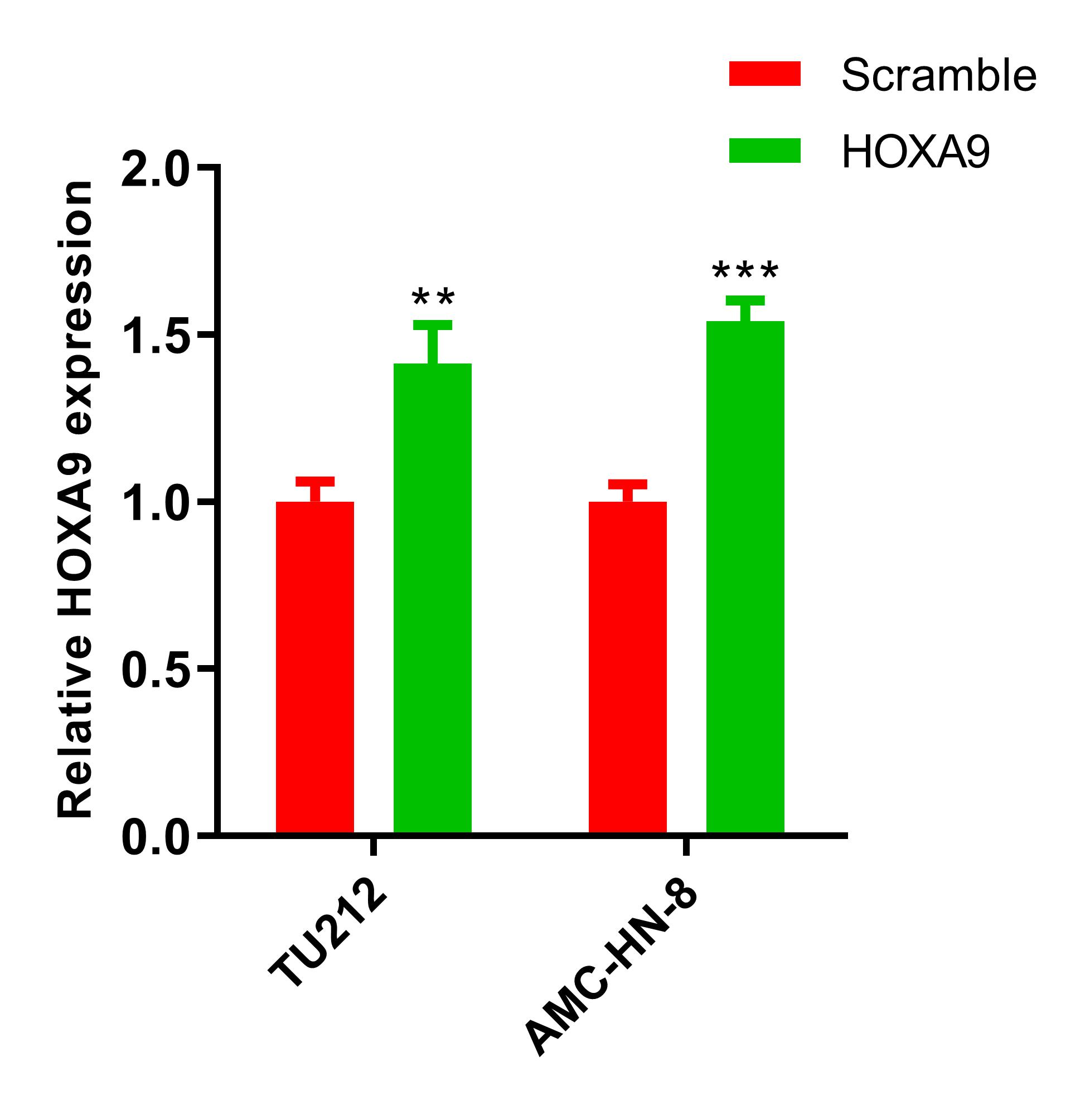
**Figure S6**

**
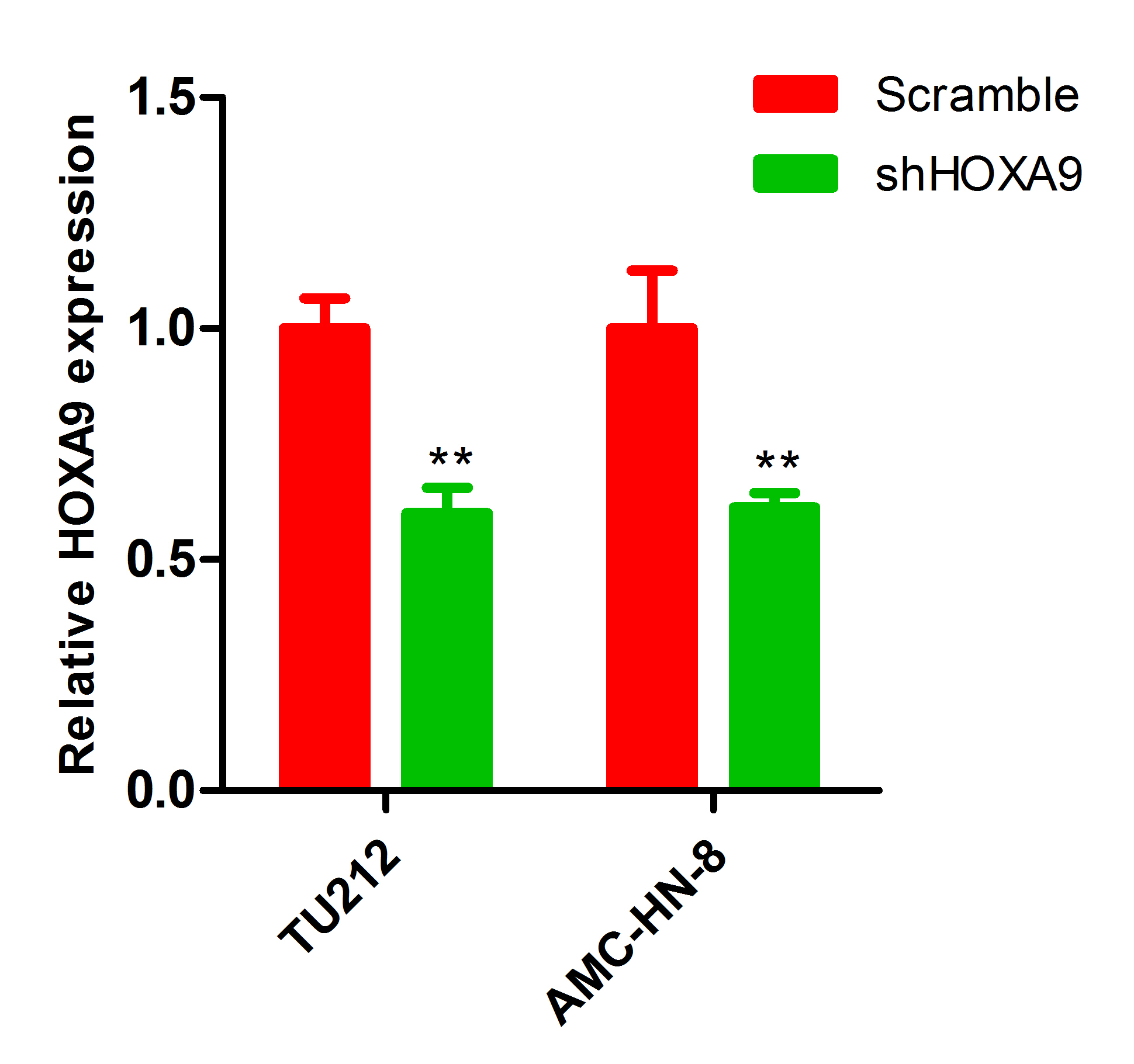
**

**A**

**B**

**Figure S6**. (A) QRT-PCR indicated the expression of HOXA9 after HOXA9 knockdown in LSCC cells. (B) QRT-PCR indicated the expression of HOXA9 after HOXA9 overexpression in LSCC cells.
