# Supplementary material for: ALKBH5‐mediated m6A modification of lncRNA KCNQ1OT1 triggers the development of LSCC via upregulation of HOXA9
Source: J Cell Mol Med. 2021 Dec 1;26(2):385–98. doi: 10.1111/jcmm.17091 (PMC8743647; doi:10.1111/jcmm.17091)
Supplement: Supplementary file 7 — Fig S7 [file JCMM-26-385-s004.doc]

**Figure S7**

**A**

**
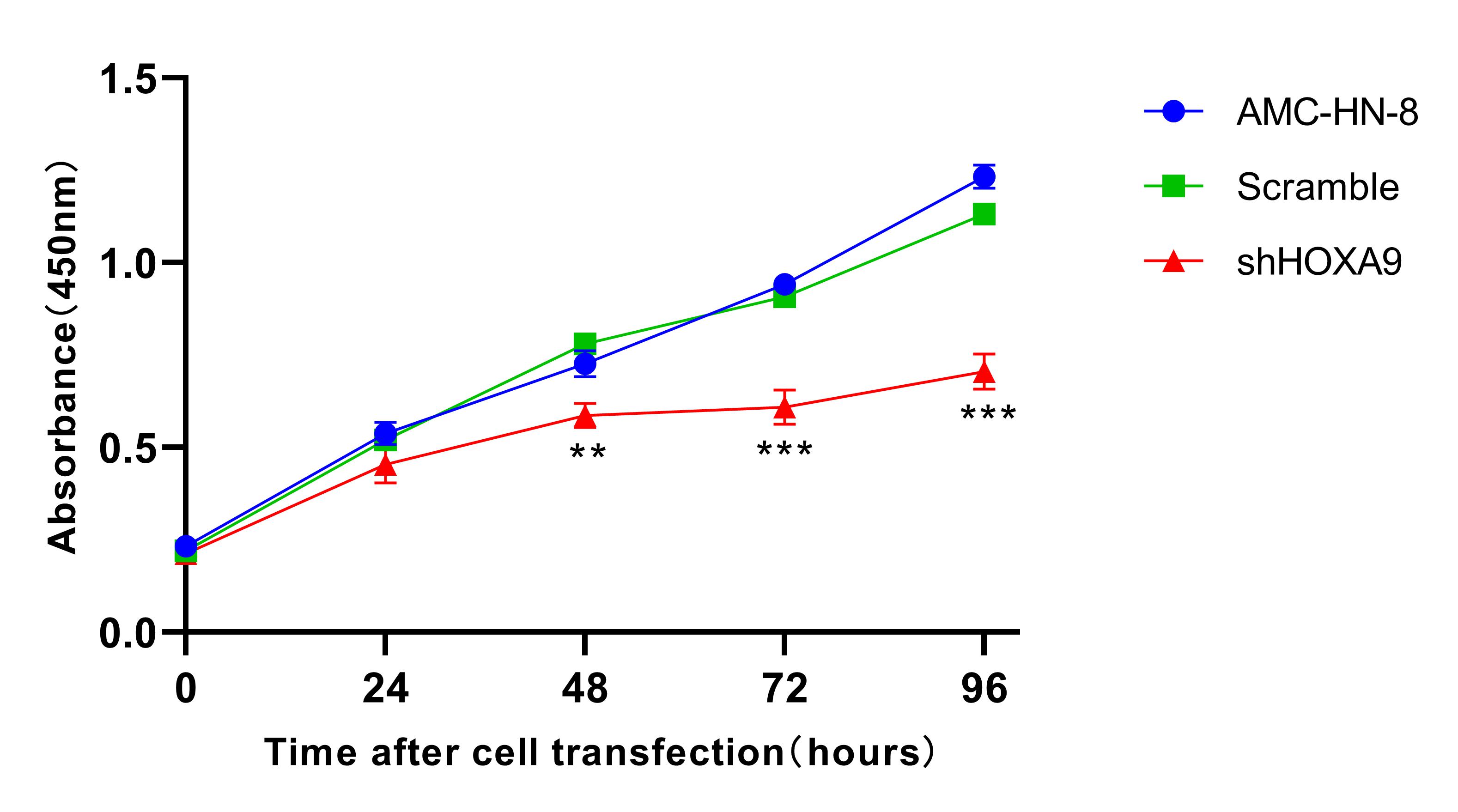

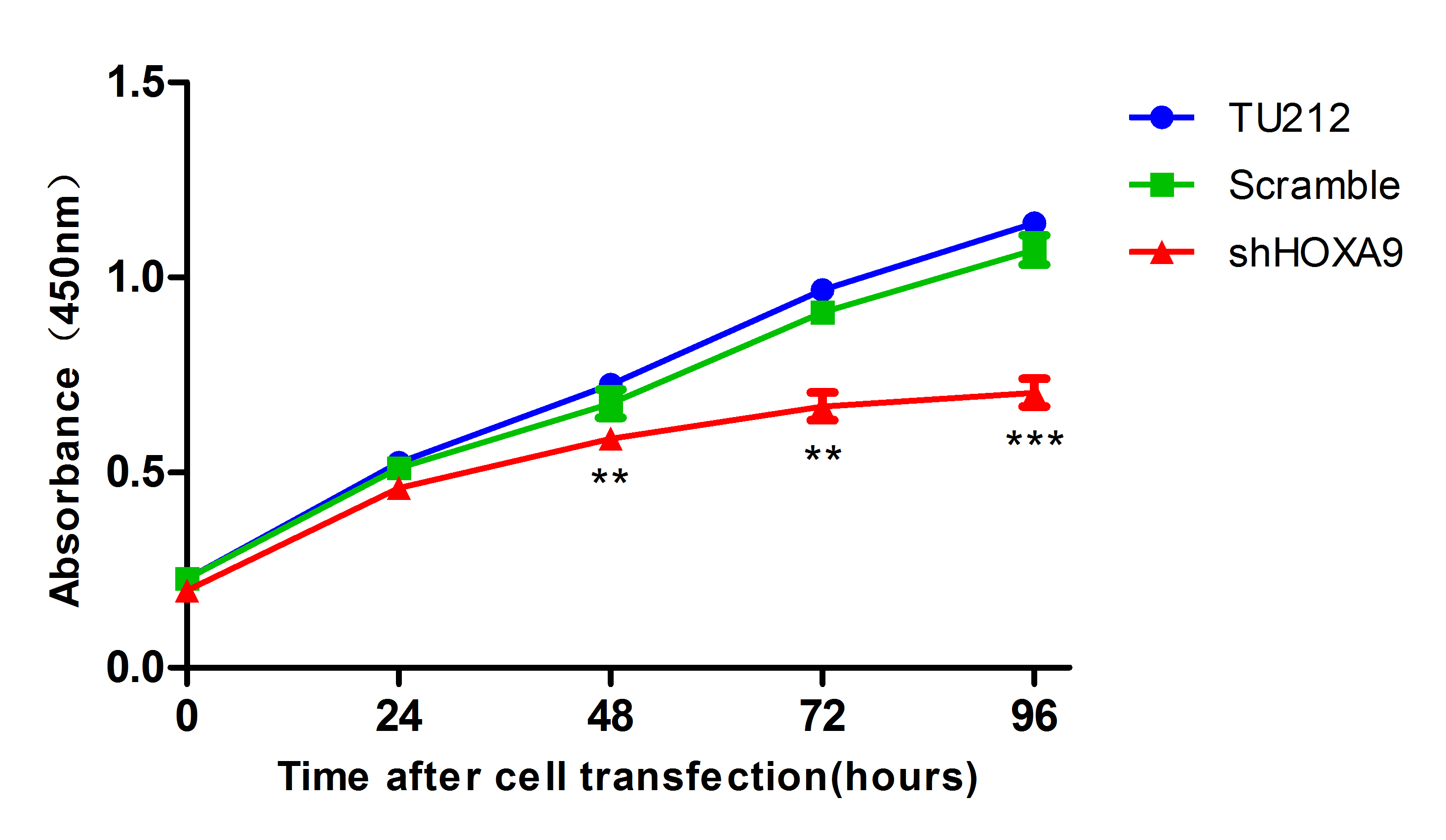
**

**
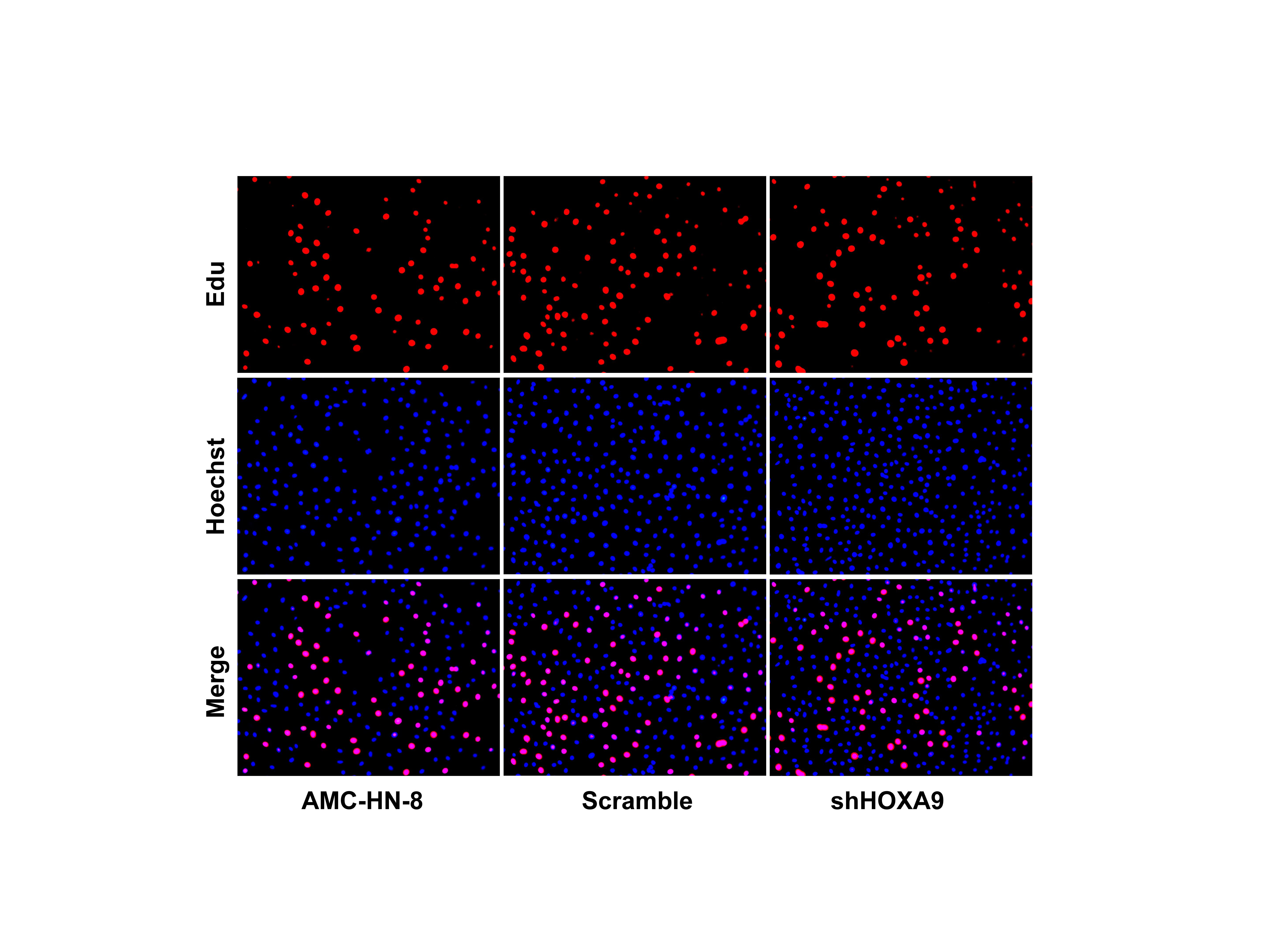

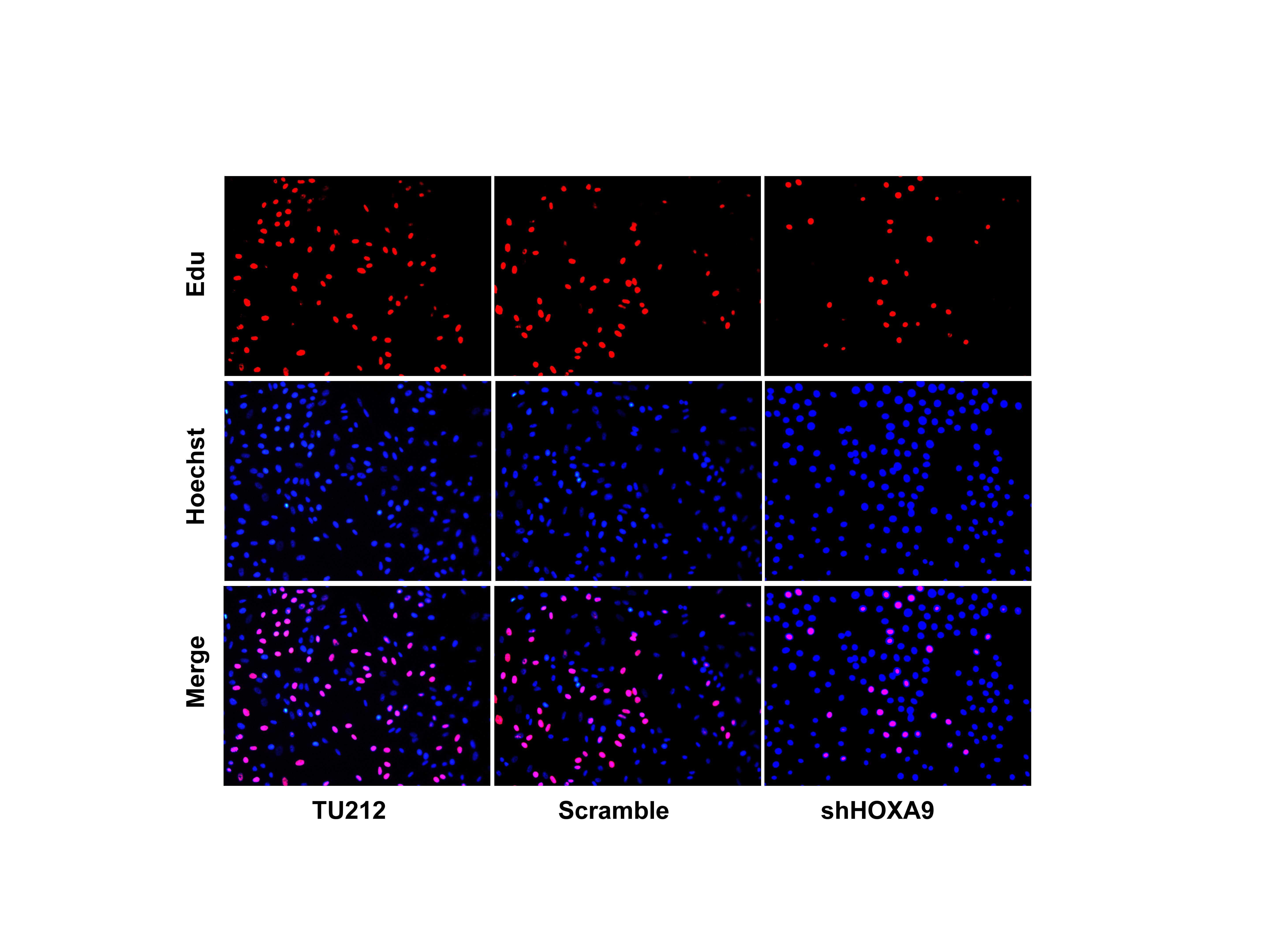
**

**B**

**
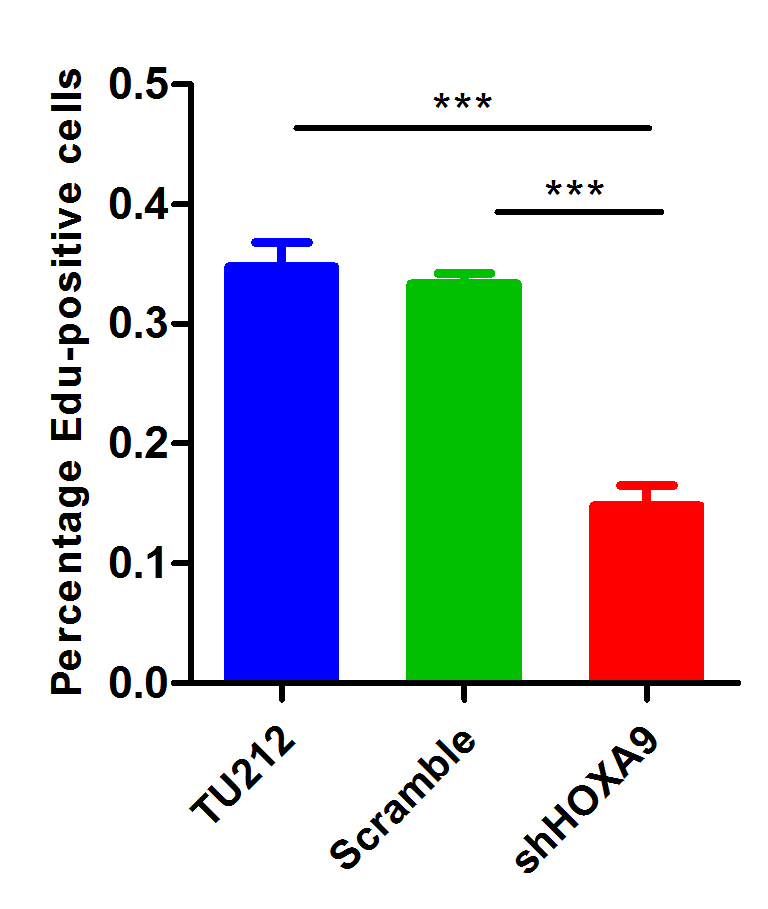

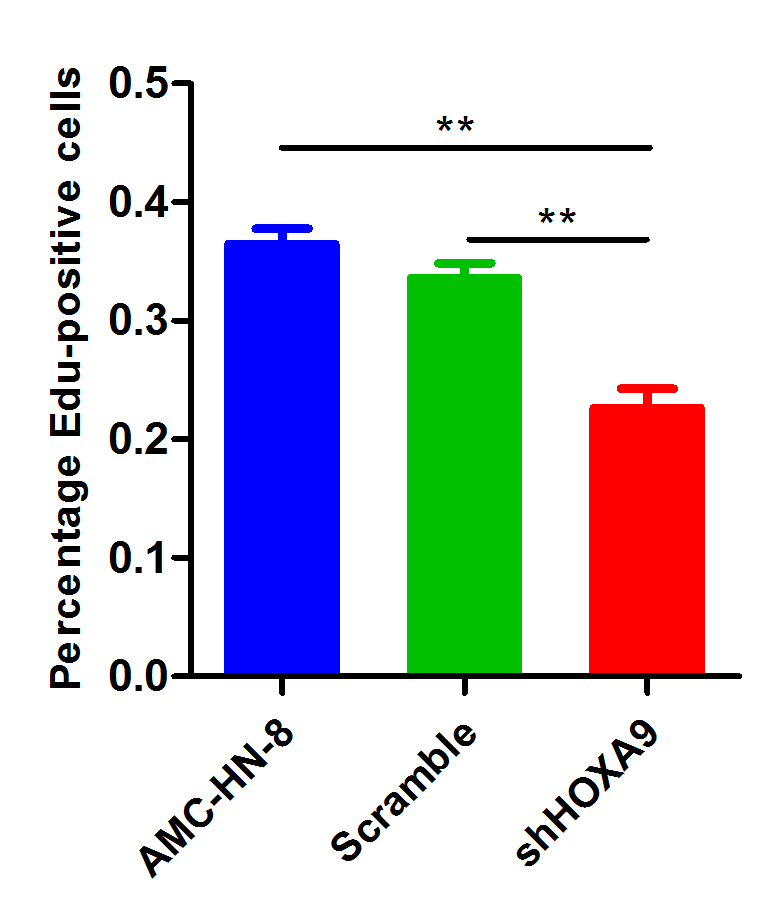
**

**
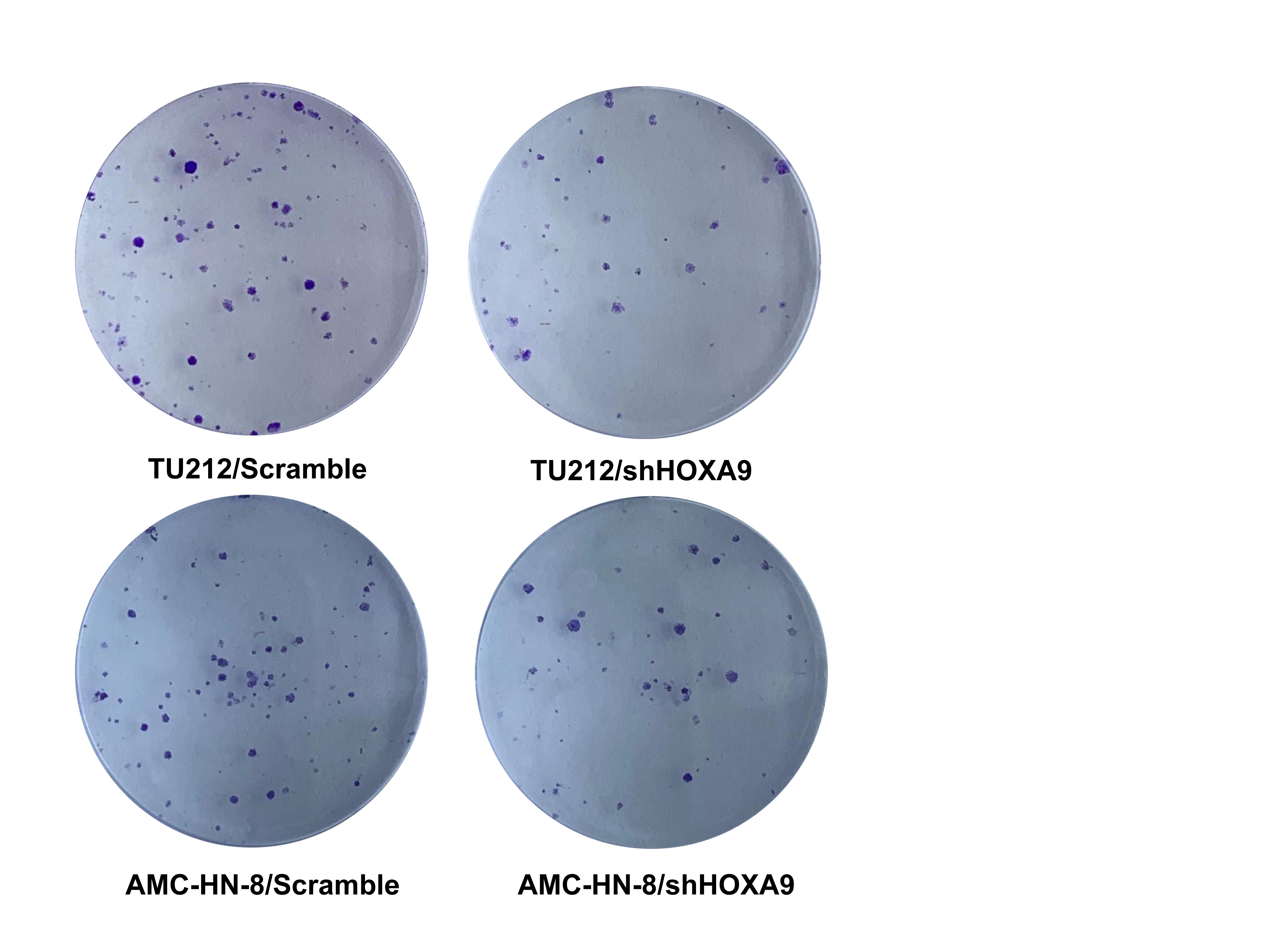
**

**C**

**
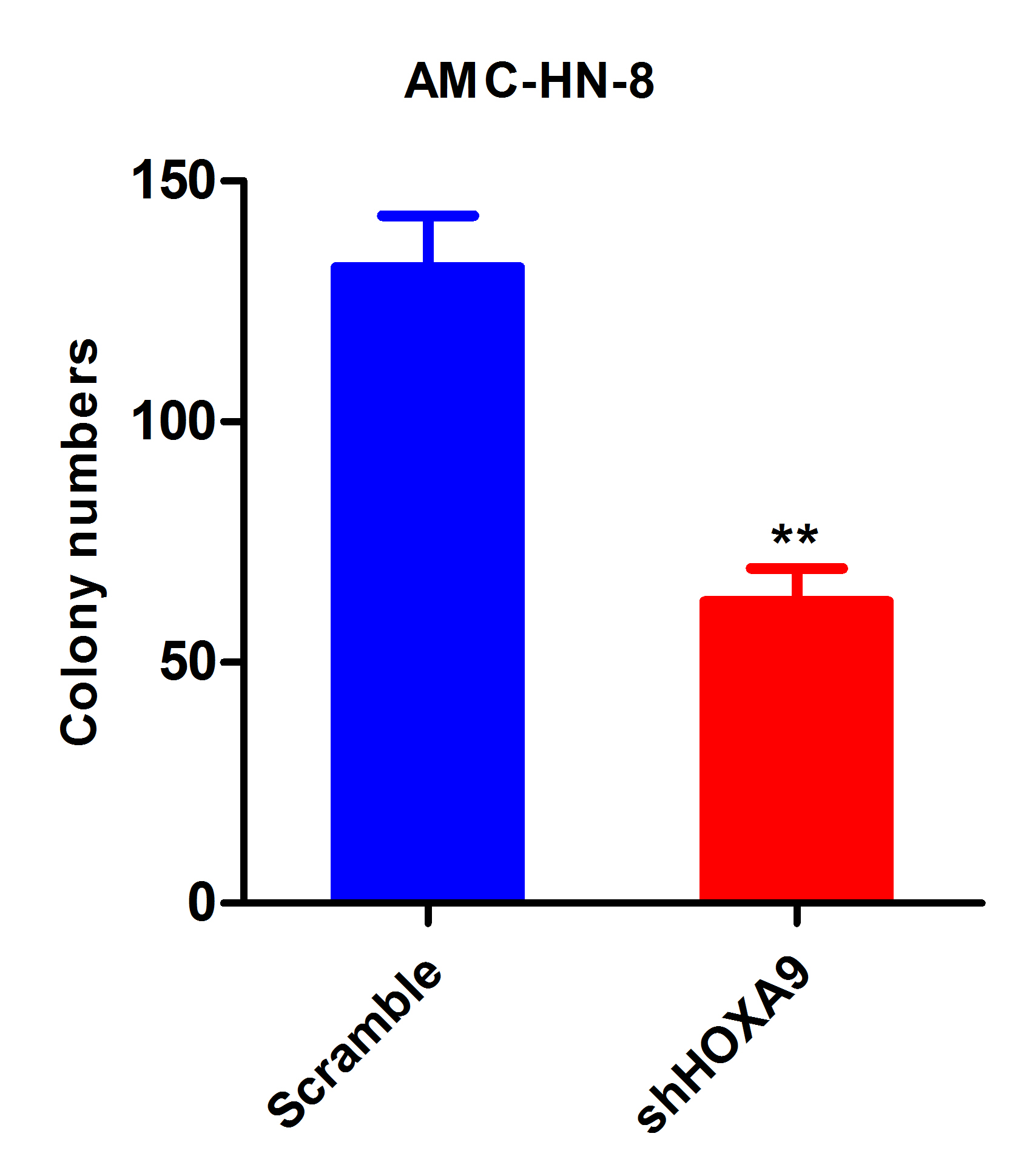

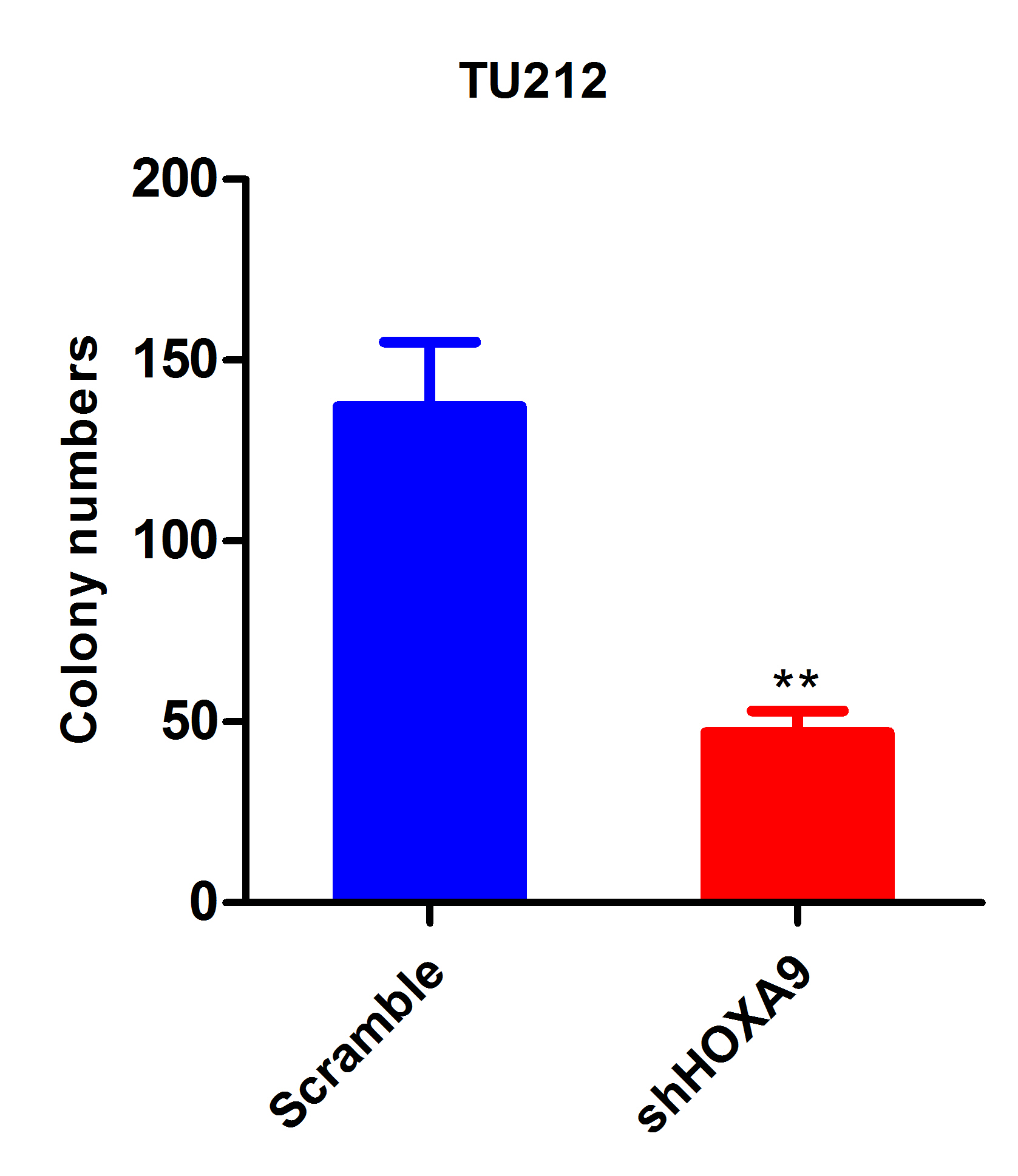
**

**
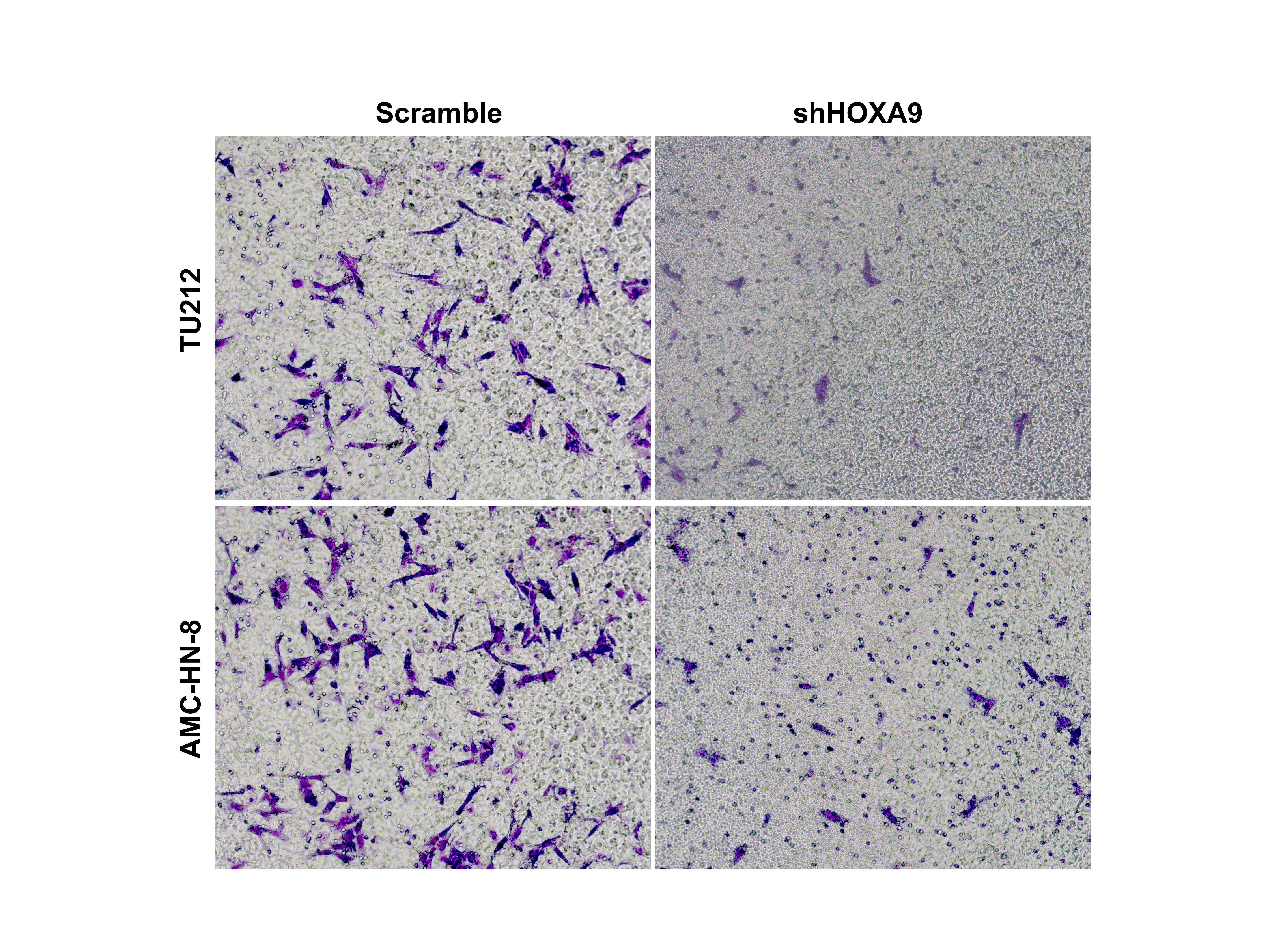

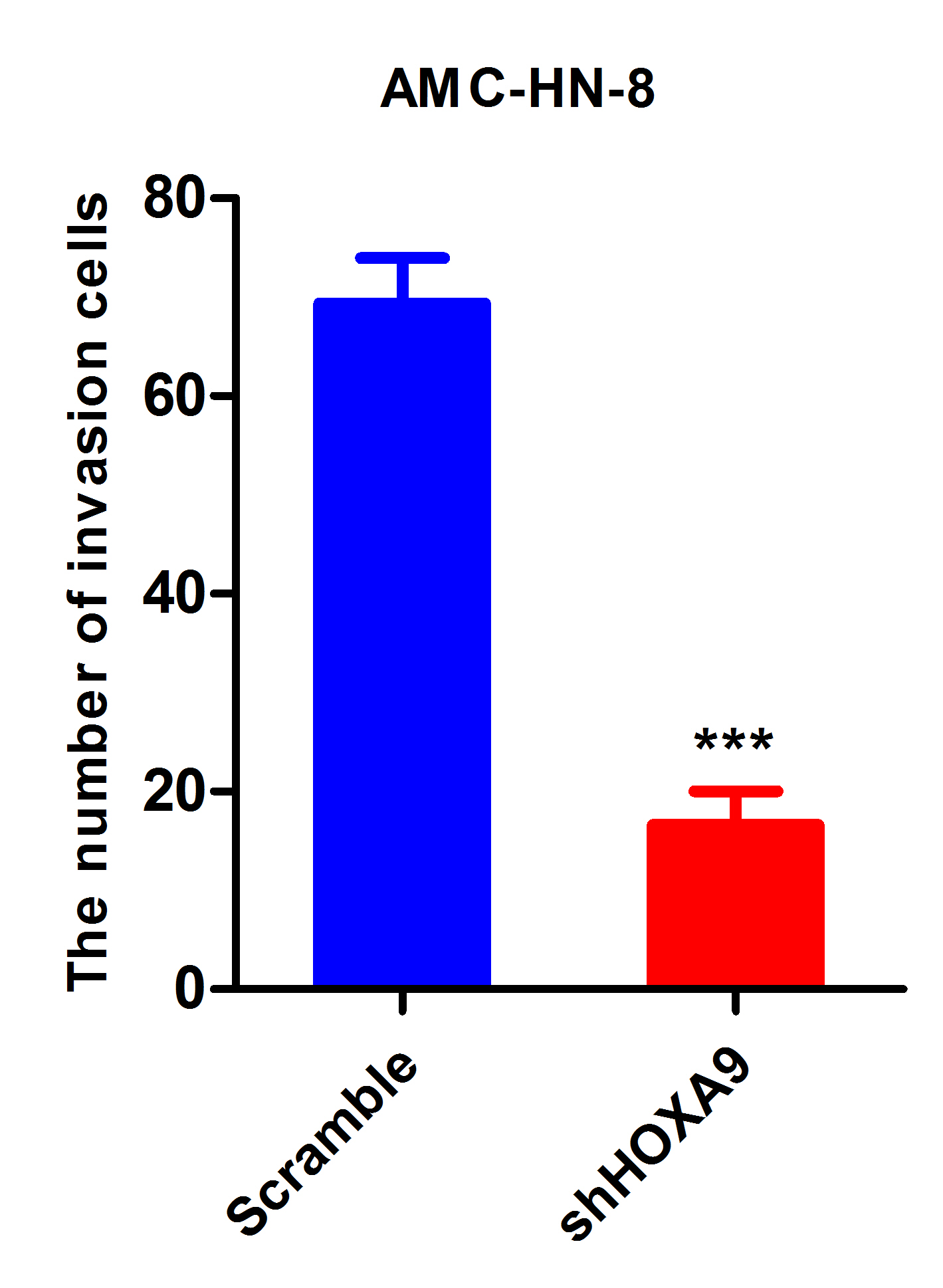

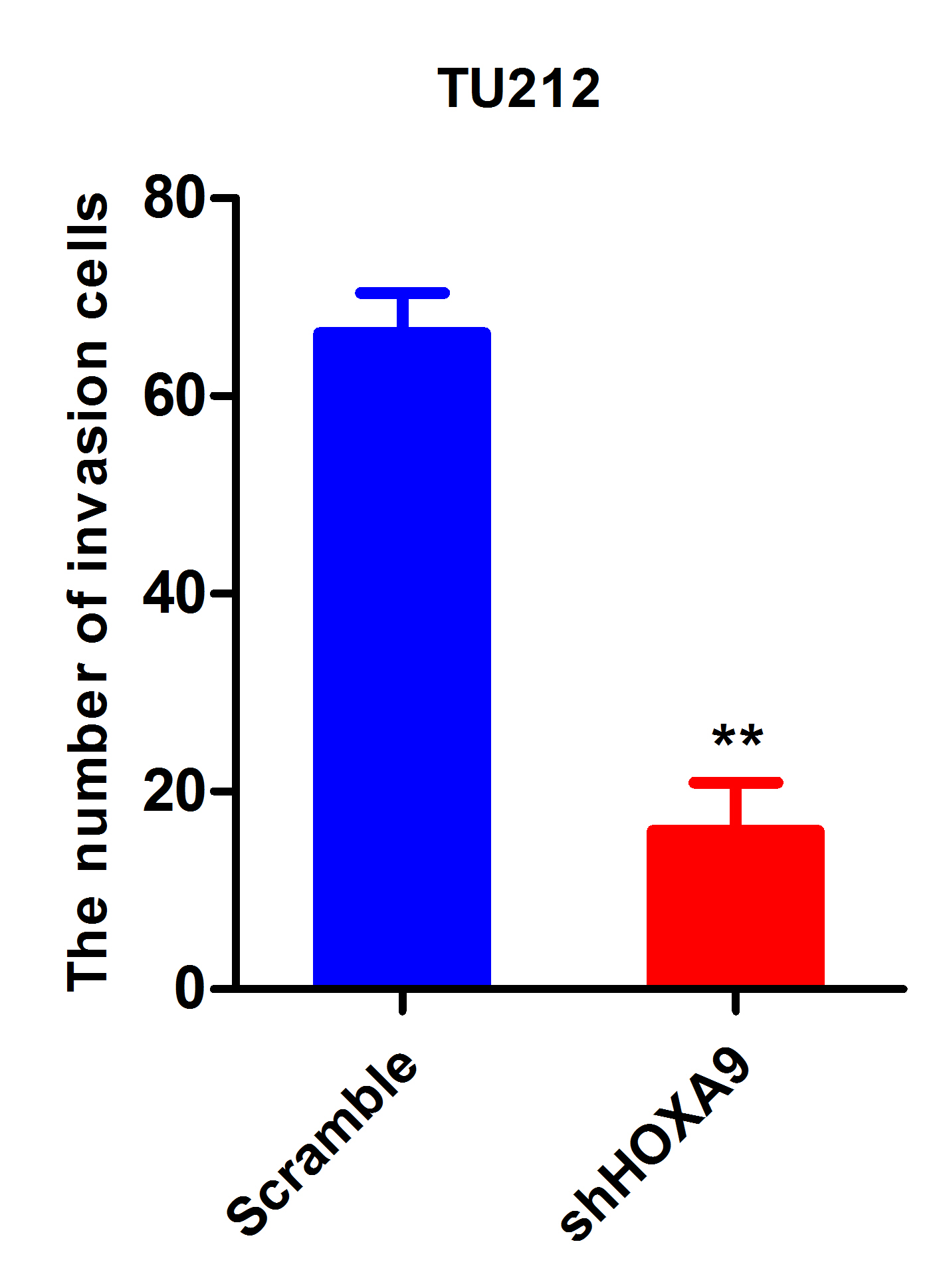
**

**D**

**
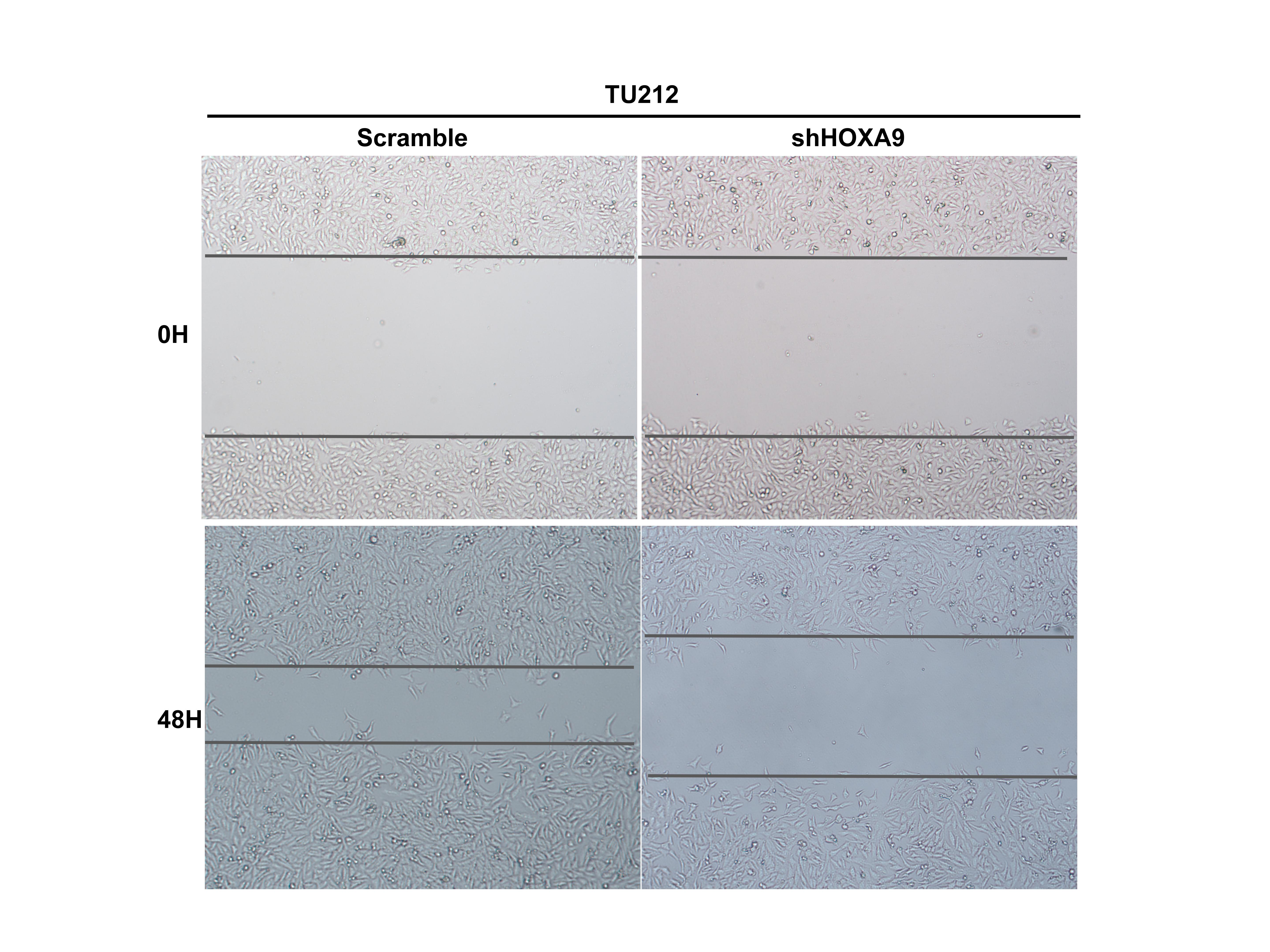

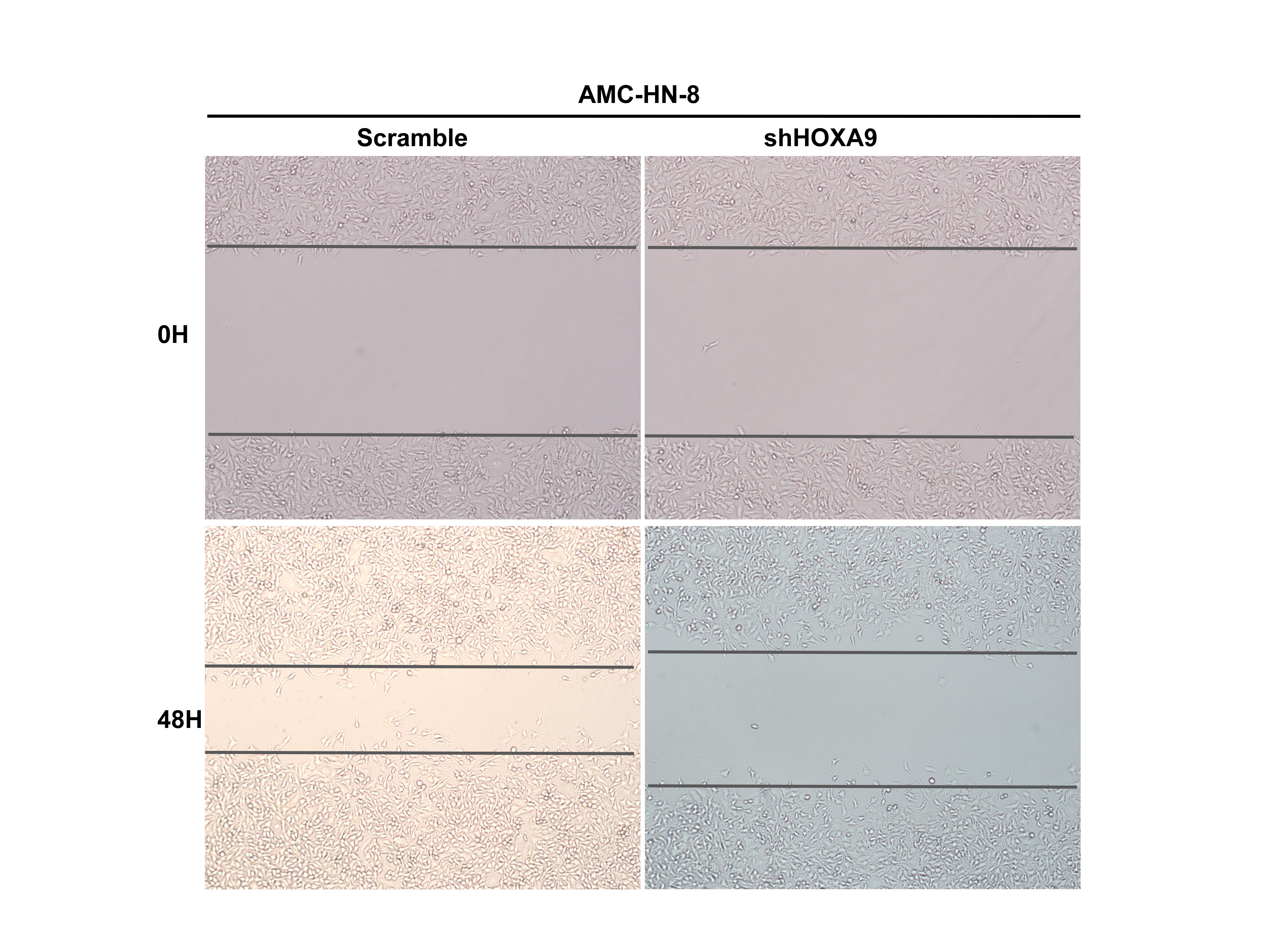

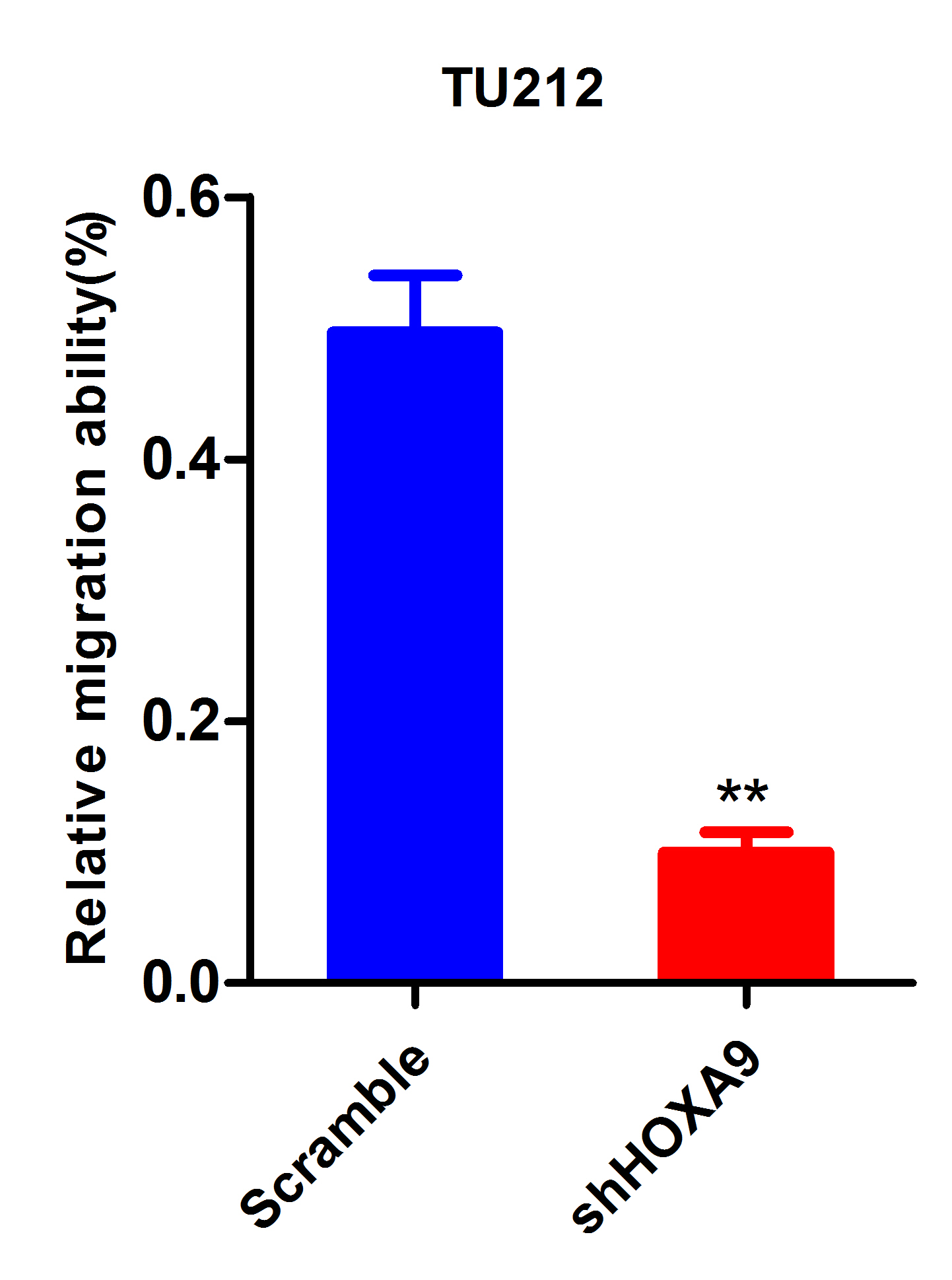
**

**
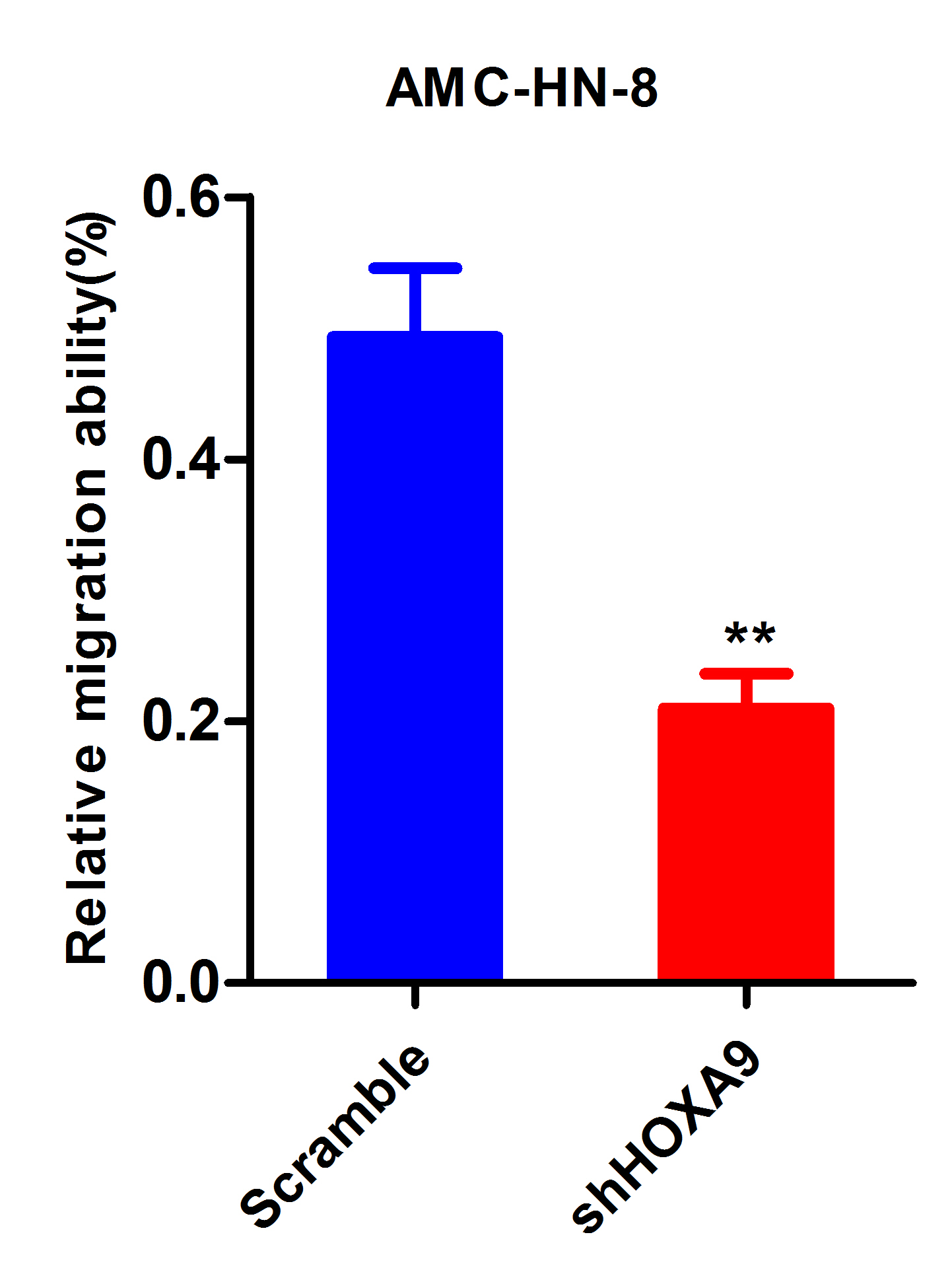
**

**E**

**Figure S7**. (A-E) CCK-8, Edu, colony formation, transwell and wound healing assays demonstrated that silencing of HOXA9 expression suppressed LSCC cells proliferation, migration and invasion ability
