# Supplementary material for: ALKBH5‐mediated m6A modification of lncRNA KCNQ1OT1 triggers the development of LSCC via upregulation of HOXA9
Source: J Cell Mol Med. 2021 Dec 1;26(2):385–98. doi: 10.1111/jcmm.17091 (PMC8743647; doi:10.1111/jcmm.17091)
Supplement: Supplementary file 8 — Fig S8 [file JCMM-26-385-s003.doc]

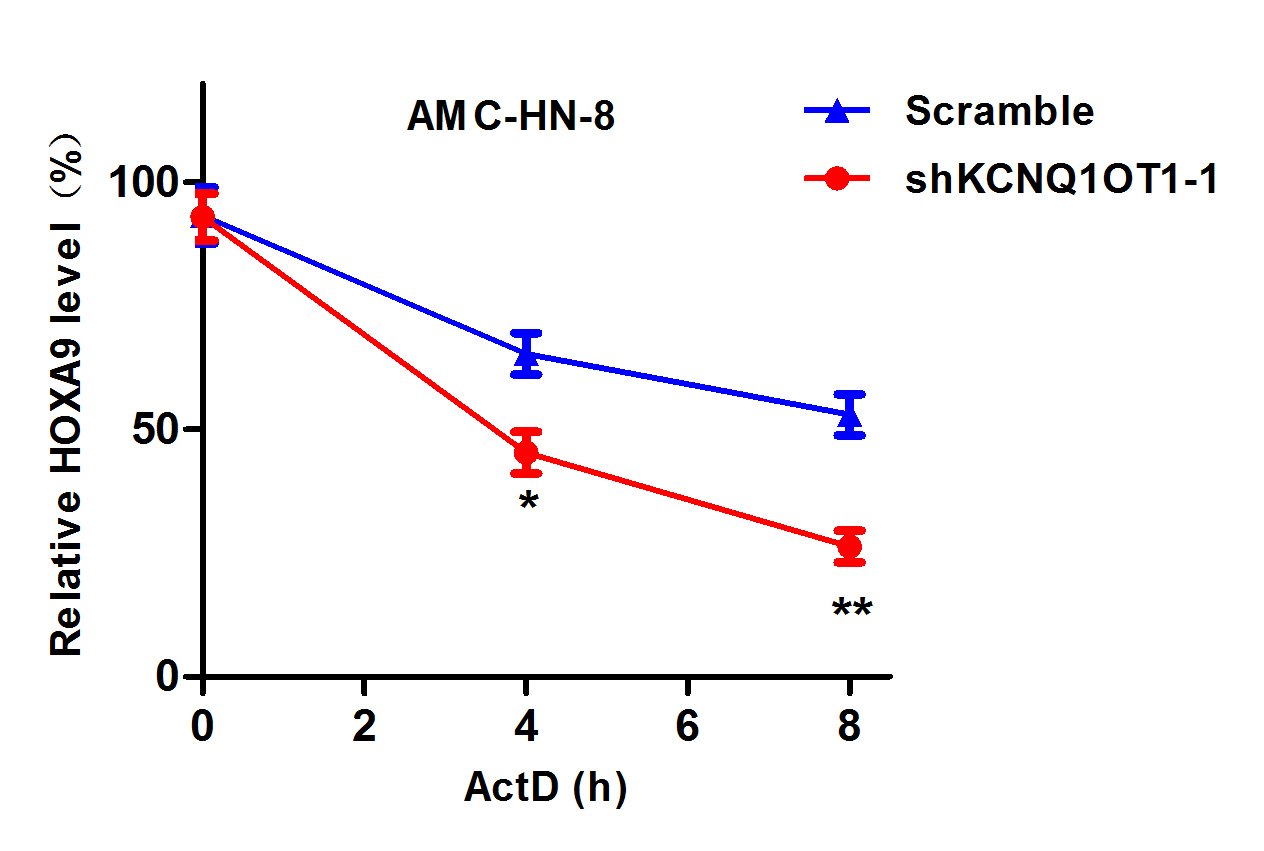

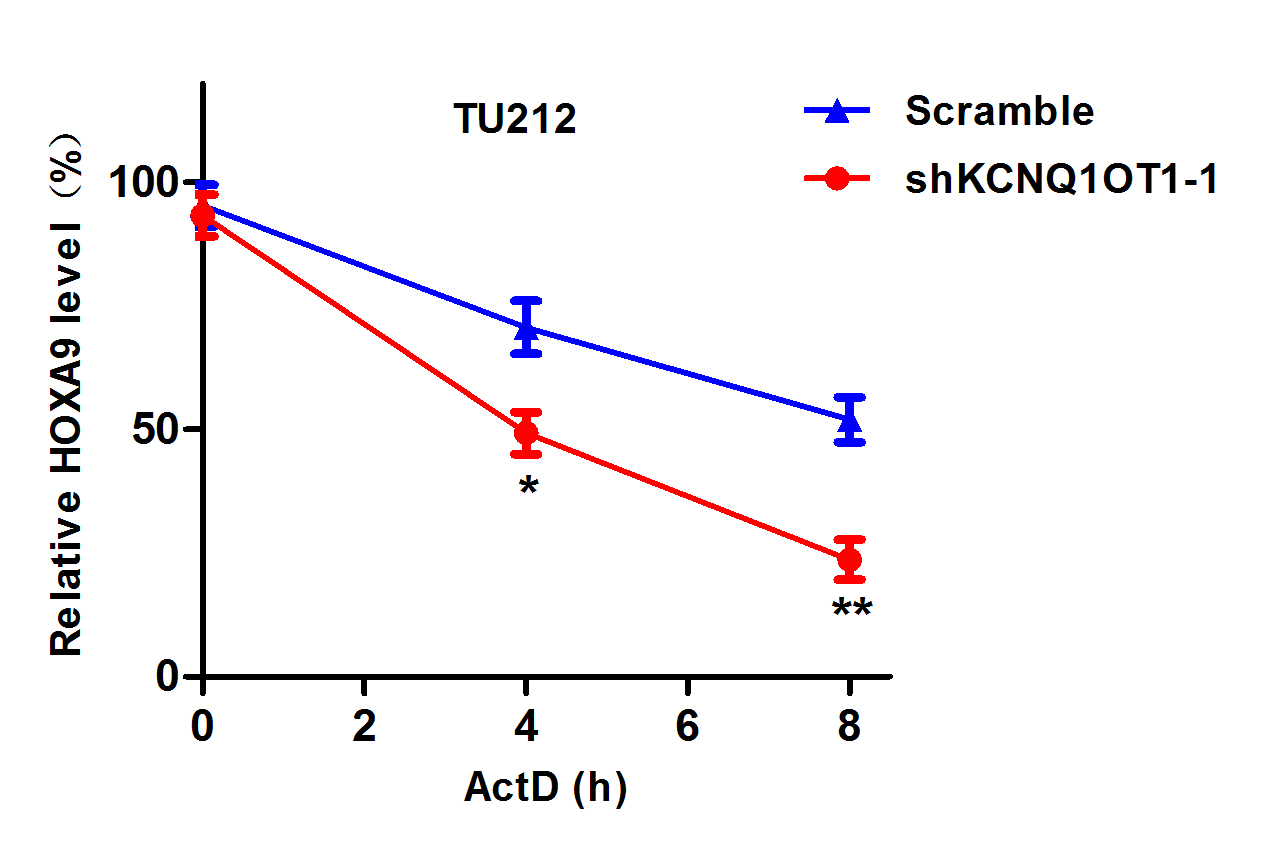
**Figure S8**

**Figure S8**. The half-life curve indicated the effect of KCNQ1OT1 knockdown on the half-life of HOXA9.
