# Supplementary material for: ALKBH5‐mediated m6A modification of lncRNA KCNQ1OT1 triggers the development of LSCC via upregulation of HOXA9
Source: J Cell Mol Med. 2021 Dec 1;26(2):385–98. doi: 10.1111/jcmm.17091 (PMC8743647; doi:10.1111/jcmm.17091)
Supplement: Supplementary file 9 — Table S1 [file JCMM-26-385-s010.doc]

**Table S1.** Primers used in the study.

| Gene | Sequences | Applications |
| --- | --- | --- |
| KCNQ1OT1 | F:5′-AGAACGGTCGCCGCGTC-3′  R:5′-ATTTATTGGCACAAAATTGT-3′ | MeRIP-qPCR  qRT-PCR |
| SPATA6L | F:5′-GTGTACCTCGGGGTCTACCTC-3′  R”5′-CCTGAATCATAATGGGGAACGC-3′ | MeRIP-qPCR |
| MECP2 | F:5′-TGACCGGGGACCCATGTAT-3′  R:5′-CTCCACTTTAGAGCGAAAGGC-3′ | MeRIP-qPCR |
| RP11-150O12.5 | F:5′-GTAACTATTCCCAGTCAGA-3′  R:5′-TACATTCTTCATACCAGGAC-3′ | MeRIP-qPCR |
| GAPDH | F:5′-TGTGGGCATCAATGGATTTGG-3′  R:5′-ACACCATGTATTCCGGGTCAAT-3′ | qRT-PCR |
| ALKBH5 | F:5′-CGGCGAAGGCTACACTTACG-3′  R:5′-CCACCAGCTTTTGGATCACCA-3′ | qRT-PCR |
| YTHDF2 | F:5′-AGCCCCACTTCCTACCAGATG-3′  R:5′-TGAGAACTGTTATTTCCCCATGC-3′ | qRT-PCR |
| HOXA9 | F:5′-TACGTGGACTCGTTCCTGCT-3′  R:5′-CGTCGCCTTGGACTGGAAG-3′ | qRT-PCR |
