# Supplementary material for: ALKBH5‐mediated m6A modification of lncRNA KCNQ1OT1 triggers the development of LSCC via upregulation of HOXA9
Source: J Cell Mol Med. 2021 Dec 1;26(2):385–98. doi: 10.1111/jcmm.17091 (PMC8743647; doi:10.1111/jcmm.17091)
Supplement: Supplementary file 10 — Table S2 [file JCMM-26-385-s001.doc]

**Table S2.** The sequences of shRNAs used in the method of cell transfection.

| shRNA | Sequences |
| --- | --- |
| Non-targeting control | 5′-GAAUACGUACCCCAUUAUA-3′ |
| shKCNQ1OT1-1 | 5′-GCCAATAGCAACTGACTAA-3′ |
| shKCNQ1OT1-2 | 5′-GCCACATCTAACACCTATA-3′ |
| shALKBH5 | 5'-CCACCCAGCTATGCTTCAGAT-3' |
| shYTHDF2 | 5′-GAACGTCAAGGTCGTGGGAAA-3′ |
| shHOXA9 | 5′-AAGTGTGAGTGTCAAGCGT-3′ |
